# End-labeled amino terminated monotelechelic glycopolymers generated by ROMP and Cu(I)-catalyzed azide–alkyne cycloaddition

**DOI:** 10.3762/bjoc.9.66

**Published:** 2013-03-25

**Authors:** Ronald Okoth, Amit Basu

**Affiliations:** 1 Department of Chemistry, Brown University, Providence, Rhode Island 02912, USA

**Keywords:** capping agent, carbohydrate, glycan, olefin metathesis, triazole

## Abstract

Functionalizable monotelechelic polymers are useful materials for chemical biology and materials science. We report here the synthesis of a capping agent that can be used to terminate polymers prepared by ring-opening metathesis polymerization of norbornenes bearing an activated ester. The terminating agent is a *cis*-butene derivative bearing a Teoc (2-trimethylsilylethyl carbamate) protected primary amine. Post-polymerization modification of the polymer was accomplished by amidation with an azido-amine linker followed by Cu(I)-catalyzed azide–alkyne cycloaddition with propargyl sugars. Subsequent Teoc deprotection and conjugation with pyrenyl isothiocyanates afforded well-defined end-labeled glycopolymers.

## Findings

Polymers functionalized with carbohydrate side chains, also referred to as glycopolymers, constitute important synthetic probes for the study of carbohydrate recognition [1–3]. Glycopolymers prepared by ring-opening metathesis polymerization (ROMP) of norbornene and cyclooctenes have been used to examine the role of glycans in such processes such as neurite outgrowth [4], bacterial motility [5], and inflammation [6]. ROMP polymers with pendant reactive functional groups, such as *N*-hydroxysuccinimide (NHS) or nitrophenyl esters, are useful materials since the pendant groups can be functionalized with various recognition elements to provide multiple final products from a common polymer intermediate in a divergent manner [7–8]. Telechelic polymers provide a means for further modifying the polymer with a variety of reporter molecules [6–7,9]. These polymers have been used as probes of biologically relevant recognition events and to functionalize surfaces [10].

Monotelechelic polymers have been obtained by the use of prefunctionalized initiators, direct end-capping with functionalized capping agents (terminating agents), and through the use of sacrificial monomers [11]. Chain termination is commonly carried out by using ethyl vinyl ether or *cis*-butene derivatives, although incomplete end-capping has been reported as a limitation with the former [12]. Notable examples include an enol ether derivative that introduces a β-trimethylsilylethoxy protected carboxylic acid at the polymer terminus [6], a *cis*-alkene pentafluorophenol ester based terminating agent that introduces an amine-reactive capping agent [13], and *cis*-olefin terminating agents that directly introduce biotin and fluorescein at the polymer terminus [14]. A recent report describes the use of a *cis*-butene phosphate derivative as a sacrificial monomer that can expose an amino terminus upon acidic hydrolysis [15]. Although these routes are attractive and efficient for obtaining monotelechelic polymers, we needed, for our purposes, a terminating agent that enables monitoring of both the capping reaction as well as subsequent polymer modifications, and that can also be removed under mild conditions for the subsequent functionalization of an amine-terminated polymer. We report here the development of the terminating agent **3**, which caps a ROMP-derived polymer with a 2-trimethylsilylethyl carbamate (Teoc) protected primary amine. The Teoc group is tolerant of subsequent polymer-side-functionalization chemistries, such as amidation and azide–alkyne click reactions. The upfield TMS ^1^H NMR resonances of the Teoc group are a useful signal for assessing the efficiency and success of post-ROMP modifications. Teoc deprotection under mildly basic conditions with tetrabutylammonium fluoride (TBAF) reveals the terminal primary amine, which can be subsequently coupled with a reporter molecule such as a dye.

**Scheme 1:**
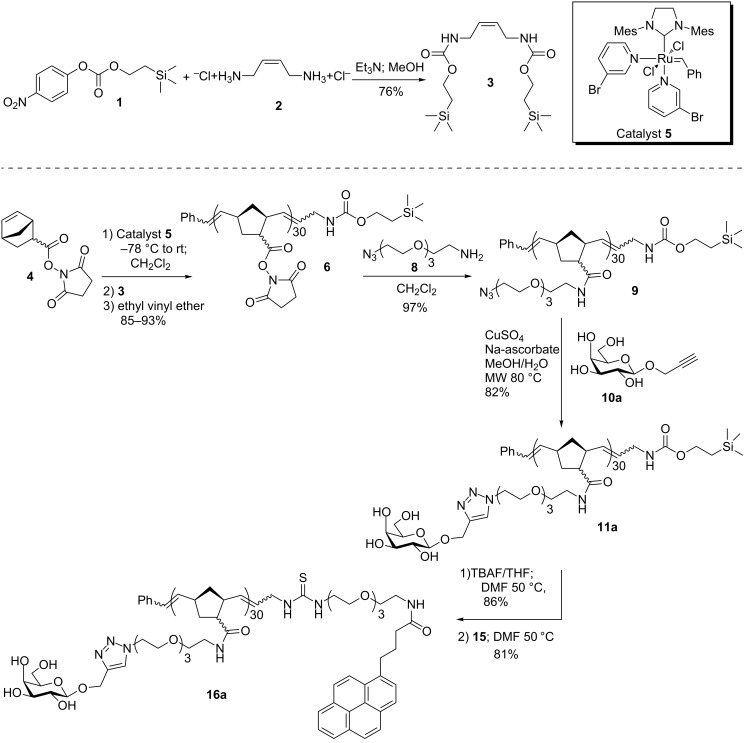
Synthesis of terminating agent **3**, ROMP and post ROMP modifications.

The terminating agent **3** was obtained by conjugation of 4-nitrophenyl (2-(trimethylsilyl)ethyl) carbonate **1** [16] and the bis-hydrochloride salt of *cis*-1,4-diamino-but-2-ene [17], **2**, as shown in Scheme 1. ROMP of bicyclo[2.2.1]hept-5-ene-2-carboxylic acid NHS ester **4** [18] was carried out by using the rapidly initiating Grubbs 3rd generation catalyst **5** [19]. A solution of monomer **4** was cooled to −78 °C, followed by addition of a solution of catalyst **5** to initiate polymerization [9]. After TLC indicated complete consumption of the monomer **4**, the reaction was treated with an excess of the terminating agent **3** for 20 minutes to provide the end-capped polymer **6**. After capping, the reaction was quenched with ethyl vinyl ether to render the catalyst inactive.

The success of the capping reaction was assessed by ^1^H NMR. Complete capping was achieved in 20 minutes, as indicated by a 5:9 integration ratio for the phenyl and trimethylsilyl protons (see Supporting Information File 1). Longer reaction times did not result in an increase in the integration of protons from the TMS group, indicating that secondary cross-metathesis events along the polymer backbone were not occurring to any significant extent in this timeframe. This is consistent with previous reports of related *cis-*butene derived terminators that required an even longer time for complete end-capping [14–15]. Treatment of longer or shorter polymers with **3** for 20 minutes was found to efficiently cap these polymers as well (see Supporting Information File 1).

Because of the labile nature of the NHS-ester substituents in polymer **6**, the stable *p*-methoxybenzylamine derivative **7** was prepared to facilitate characterization of the polymer by MALDI–ToFMS analysis (see Supporting Information File 1). Analysis of the MALDI spectra of polymer **7** showed incorporation of the end groups and the uniform interpeak distance confirmed the uniform incorporation of the Teoc terminus. The molecular data for **7** obtained by MALDI was in agreement with that obtained from GPC and NMR analysis of polymer **6** (see Supporting Information File 1).

**Figure 1:**
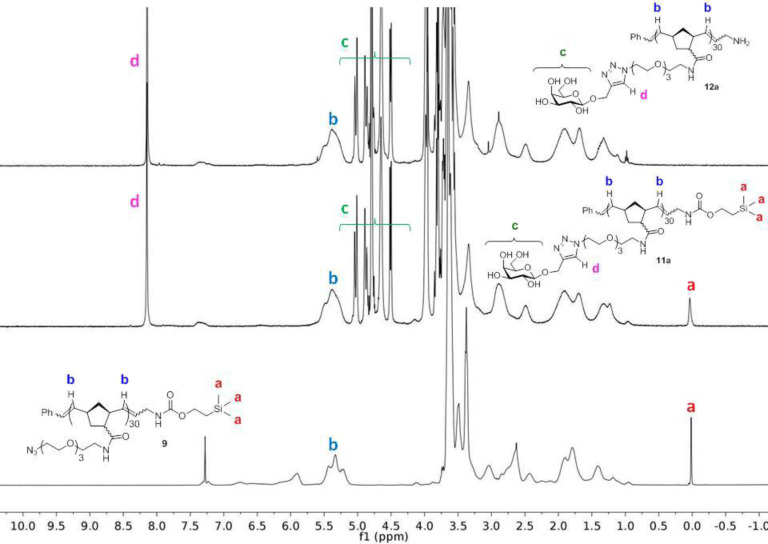
Overlay of ^1^H NMR spectra of azido-polymer **9** (bottom) showing TMS protons – **a** and olefinic protons – **b**. Galactosyl glycopolymer **11a** (middle) showing introduction of sugar protons – **c** and triazole protons – **d**. Amino-terminated galactosyl glycopolymer **12a** (top) showing disappearance of TMS protons.

Due to its high conversion and functional-group tolerance the copper(I)-promoted azide–alkyne click reaction (CuAAC) has become a powerful post-polymerization modification reaction. Post-polymerization modification of macromolecules by click reaction can be accomplished in two ways; (i) a macromolecule bearing alkyne groups is reacted with an azido-monomer or (ii) a macromolecule bearing azide groups is reacted with a monomer bearing a terminal alkyne [20–21]. For our purposes the later method was attractive as we could follow the reaction by monitoring the disappearance of the azide asymmetric stretch at 2100 cm^−1^ by IR spectroscopy. As shown in Scheme 1, reaction of the ROMP polymer **6** with excess amino*–*azide linker **8** [22] (2 equivalents for every NHS substituent in polymer **6**) afforded azido-polymer **9**. Quantitative displacement of NHS by **8** was based on complete disappearance of succinimide protons at 2.80 ppm in the ^1^H NMR spectrum (Figure 1, bottom spectrum). Subsequent modifications on the polymer (see below) confirmed that all of the NHS esters had indeed been displaced.

CuAAC between propargyl β-D-galactoside **10a** [23] and azido polymer **9** was achieved in 10 minutes by a microwave-assisted protocol to give galactosyl polymer **11a**. We also prepared the mannosylated glycopolymer **11b** in a similar manner (see Supporting Information File 1). The progress of the CuAAC was assessed by monitoring the appearance of the triazole peak at 8.15 ppm in the ^1^H NMR spectra of **11a** (Figure 1, middle spectrum). Furthermore, the IR spectra showed complete disappearance of the azide asymmetric stretch at 2100 cm^−1^ upon completion of the reaction (see Supporting Information File 1). The ratio of the triazole to TMS protons observed in the ^1^H NMR spectrum (ca. 40:9) is consistent with complete displacement of all NHS-ester substituents by the amino*–*azide linker **8** in the previous step. Attempts to carry out the CuAAC with propargyl 3’-sulfo-β-D-galactoside (propargyl SGal) **10c** under the standard conditions resulted in only 50% conversion as determined by ^1^H NMR. Complete conversion was only obtained upon the use of the Cu(I) stabilizing ligand tris(3-hydroxypropyltriazolylmethyl)amine [24] (THPTA) to afford the polymer **11c**.

Treatment of the glycopolymers **11** with TBAF yielded the amino-terminated monotelechelic glycopolymers **12**. The complete disappearance of the TMS protons in ^1^H NMR (Figure 1, top spectrum) confirmed the successful and complete deprotection. Subsequent conjugation of **12** with the pyrene isothiocyanate **15**, prepared from the NHS ester of pyrene butyric acid **13** via intermediate **14**, afforded the pyrene-end-labeled glycopolymers **16**. The extent of pyrene labeling was determined by UV–vis spectroscopy and found to be essentially quantitative for the galactosyl and mannosyl polymers. The SGal polymer **12c** only underwent 60% labeling under these conditions.

In conclusion we have presented here a facile method for accessing monotelechelic ROMP polymers and demonstrated that subsequent post-polymerization modifications can be assessed accurately by a general method such as ^1^H NMR. This method allows access to well-defined glycopolymers and enables subsequent polymer terminus functionalization upon deprotection of the Teoc moiety. The polymer **9** can be functionalized with a variety of groups on the side-chain given the generality of the CuAAC reaction, and should be of utility for the synthesis of a variety of functionalized monotelechelic polymers. We are currently evaluating the polymers **16a**–**c** as probes for the carbohydrate–carbohydrate interaction between galactose and 3-sulfogalactose [23], and those studies will be reported in due course.

## Supporting Information

The Supporting Information contains detailed experimental procedures and characterization data for small molecules and polymers, including polymer GPC and MALDI data, and NMR spectra.

File 1 Experimental procedures and characterization data.
