# Supplementary material for: End-labeled amino terminated monotelechelic glycopolymers generated by ROMP and Cu(I)-catalyzed azide–alkyne cycloaddition
Source: Beilstein J Org Chem. 2013 Mar 25;9:608–12. doi: 10.3762/bjoc.9.66 (PMC3628285; doi:10.3762/bjoc.9.66)
Supplement: File 1 — Experimental procedures and characterization data. [file Beilstein_J_Org_Chem-09-608-s001.pdf]

Supporting Information

for

End-labeled amino terminated monotelechelic glycopolymers generated by ROMP and Cu(I)-catalyzed azide–alkyne cycloaddition

Ronald Okoth and Amit Basu*

Address: Department of Chemistry, Brown University, Providence, Rhode Island 02912, USA

Email: Amit Basu* - abasu@brown.edu

* Corresponding author

Experimental procedures and characterization data.

page

1. Experimental procedures……………………...…………………………………….…...…....S2
2. References……………………………………………………………..………......S15
3. MALD–ToF analysis……………...…………………………………………...……....S16
4. GPC analysis.……………………………………………………….....………..… S17
5. Capping efficiency table…………..…………………………...……………....…....S18
6. IR spectra of poymers………………………………………………………..…...…S19
7. Determination of extent of pyrene end-labeling ……………………………….…..….S20
8. Teledyne ISCO COMBI Flash R_f chromatograms...……………..…………………….…S24
9. NMR spectra…………………………………………………………………….…...S27

1. Experimental procedures

General procedures and materials. Dry solvents were obtained from a commercially available solvent purification system based on protocols reported by Grubbs [1]. All reactions were carried out under nitrogen atmosphere unless otherwise stated. Automatic flash chromatography was performed using Teledyne ISCO COMBI Flash Rf instrument using Redi Sep Rf silica columns (24 g and 40 g) and self packed silica cartridges. Water, pyridine and DMF were evaporated using a Centrifan PE from Modular SFC unless otherwise stated. Dialysis was done using 6 Spectra/Por regenerated cellulose dialysis membrane 1000 MWCO from Spectrum. Concentration by centrifugal filtration was done using Amicon Ultra centrifugal filters (regenerated cellulose 3000 MWCO) filter devices in a Sorvall ST 16 centrifuge from Thermo Scientific operated at 4000x g. Centrifugation of the precipitated polymers **6** was done using an Adams physician's compact centrifuge. NMR spectra were recorded in BRUKER 400 MHz or 300 MHz instruments for ^{1}H NMR, 100 MHz or 75 MHz for ^{13}C NMR and referenced at 2.05 ppm for d_6-acetone, 4.79 ppm for D_2O, 7.26 ppm for $CDCl_3$, and 7.16 ppm for C_6D_6 in ^{1}H NMR and 128.06 ppm for C_6D_6 77.16 ppm for $CDCl_3$ in ^{13}C NMR. ^{1}H NMR for all polymer products were obtained with a delay time D[1] of 5 seconds. MALDI-ToF spectra were obtained using a VOYAGER-DE PRO bio-spectrometry workstation instrument using super DHB matrix (2,5-dihydroxybenzoic acid doped with 2-hydroxy-5-methoxybenzoic acid). GPC of polymers **6** were obtained using AM GPC Gel column with linear 10 µm porosity from American Polymer Standards Corporation mounted in a Hewlett Packard 1090 liquid chromatography machine with a UV detector. Agilent 8453 UV–visible spectroscopy

system was used to obtain UV–vis spectra of **15** and pyrene-labeled glycopolymers **16** DMF solutions in a 1 cm quartz cell.

TLC stains were prepared as follows; $KMnO_4$ (3 g $KMnO_4$, 20 g K_2CO_3, 5 mL of 5% NaOH, 300 mL H_2O); anisaldehyde (9.2 mL anisaldehyde, 3.75 mL AcOH, 338 mL 95% EtOH, 12.5 mL conc. H_2SO_4) TLC Silica Gel 60 F_{254} plates from EMD Chemicals were used.

4-Nitrophenyl (2-(trimethylsilyl)ethyl) carbonate (**1**) was prepared by a slight variation of the protocol reported by Rosowsky [2]. *cis*-1,4-Diamino-but-2-ene•2HCl salt (**2**) was prepared as reported by Greenaway [3]. Bicyclo[2.2.1]hept-5-ene-2-carboxylic acid *N*-hydroxysuccinimide ester (**4**) was prepared by a slight variation of the protocol reported by Kiessling [4]. Catalyst **5** was prepared as reported by Grubbs [5]. 1,11-Diazido-3,6,9-trioxaundecane was prepared as reported by Schwabacher [6]. Propargyl galactoside **10a**, propargyl mannoside **10b** and propargyl 3' sulfogalactoside **10c** were prepared as reported by Basu [7]. 2-(2-(2-Aminoethoxy)ethoxy)ethanol tris(3-hydroxy-propyltriazolylmethyl)amine (THPTA) ligand was prepared as reported by Finn [8]. *N*-(1-pyrene)butyryloxysuccinimide **13** was prepared as reported by Wu [9].

4-nitrophenyl (2-(trimethylsilyl)ethyl) carbonate (**1**) [2]

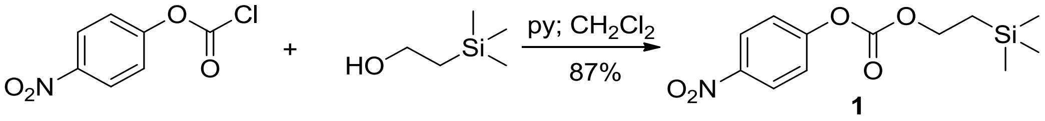

To a solution of 4-nitrophenyl chloroformate (6.00 g, 30 mmol) in 40 mL dry CH_2Cl_2 at 0 °C was added a solution of 2-trimethylsilylethanol (3.8 mL, 3.14 g, 26.5 mmol) and pyridine (2.2 mL, 2.13 g, 26.5 mmol) in 20 mL dry CH_2Cl_2 via a syringe dropwise over 10 min, and the reaction mixture was allowed to warm to room temperature. After 24 h the reaction mixture was washed four times with 100 mL saturated $NaHCO_3$ and twice with brine, and then dried over Na_2SO_4. The CH_2Cl_2 solution was concentrated in vacuo, and the pale yellow oily residue was purified by automatic flash chromatography (EtOAc:Hexanes 1:9 to 4:6) to give **1** (6.52 g, 23 mmol) 87% as pale yellow oil which solidified upon refrigeration. TLC (Silica – EtOAc:hexanes 1:3 – UV) R_f 0.69. ^{1}H NMR (400 MHz, $CDCl_3$) δ 8.30–8.23 (m, 2H) 7.40–7.32 (m, 2H) 4.44–4.29 (m, 2H) 1.21–1.09 (m, 2H) 0.08 (s 9H) ^{13}C NMR (100 MHz, $CDCl_3$) δ 155.8, 152.6, 145.4, 125.4, 122.0, 68.4, 17.7, −1.4 HRMS (FAB) calculated for $C_{12}H_{17}NO_5Si$ $[M + Na]^+$ 306.0774 found 306.0780.

cis-Bis(2-(trimethylsilyl)ethyl) but-2-ene-1,4-diyldicarbamate (**3**)

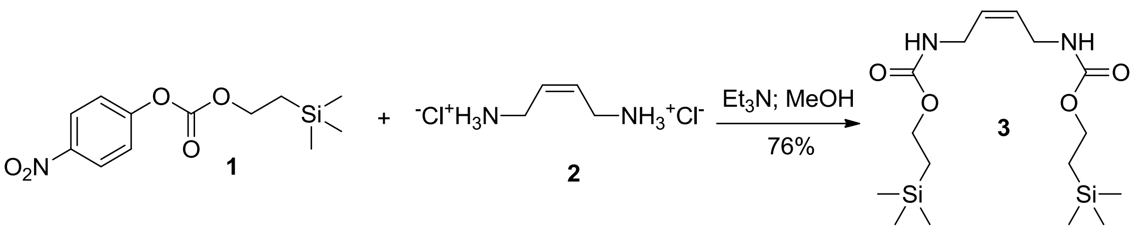

cis-1,4-Diamino-but-2-ene•2HCl salt **2** [3] (1.17 g, 7.35 mmol) was dissolved in 50 mL dry MeOH, 2.1 mL Et_3N (1.50 g, 14.7 mmol) was added to the flask followed by **1** (4.17 g, 14.7 mmol). The reaction was stirred at room temperature overnight. The MeOH

was evaporated, and the yellow oily residue was dissolved in 100 mL CH_2Cl_2 and washed with saturated $NaHCO_3$ until the aqueous layer was clear. The CH_2Cl_2 layer was then washed with 100 mL brine, dried over Na_2SO_4, and concentrated in vacuo. The light brown oily residue was purified by column chromatography (silica, 1:3 EtOAc: Hexanes) to give **3** (2.16 g, 5.77 mmol) 76% as a clear oil. TLC (silica–EtOAc:hexanes 1:3 – $KMnO_4$ stain) R_f 0.55. ^{1}H NMR (400 MHz, $CDCl_3$) δ 5.54 (t, J = 4.6 Hz, 2H) 5.03 (bs, 2H) 4.17 (t, J = 8.4 Hz, 4H) 3.82 (t, J = 5.7 Hz, 4H) 0.97 (t, J = 8.4 Hz, 4H) 0.02 (s, 18H). ^{13}C NMR (75 MHz, $CDCl_3$) δ 156.9, 128.9, 63.2, 37.5, 17.9, −1.3 HRMS (FAB) calculated for $C_{16}H_{34}N_2O_4Si_2$ $[M + Na]^+$ 397.1955 found 397.1966.

Bicyclo[2.2.1]hept-5-ene-2-carboxylic acid *N*-succinimide ester (**4**) [4]

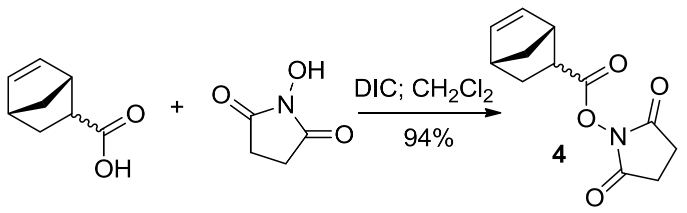

To a solution of 5-norbornene-2-carboxylic acid (3.49 g, 25.2 mmol) and *N*-hydroxysuccinimide (3.48 g, 30.3 mmol) in 50 mL dry CH_2Cl_2 at 0 °C was added 4.7 mL *N,N'*-diisopropylcarbodiimide (DIC) (3.83 g, 30.3 mmol) dropwise via a syringe, and the reaction was stirred overnight. The white precipitate was filtered and washed with 10 mL CH_2Cl_2 twice, and the filtrate was concentrated and purified by automatic flash chromatography (EtOAc:DCM 1:9 to 6:4) to give **4** (5.55 g, 23.6 mmol) in 94% yield as a white solid. TLC (Silica–DCM - $KMnO_4$ stain) R_f = 0.34. ^{1}H NMR (400MHz, $CDCl_3$) δ 6.27–6.09 (m, 2H) 3.40 (s, 1H) 3.24 (dt, J = 9.1, 3.8 Hz, 1H) 2.99 (s, 1H) 2.81 (bs, 4H) 2.51 (ddd, J = 9.0, 4.6, 1.4 Hz, 1H) 2.09–1.93 (m, 1H) 1.56–1.23 (m, 3H); ^{13}C NMR

(100 MHz, $CDCl_3$) δ 170.1, 169.4, 138.7, 138.3, 135.4, 132.3, 49.8, 47.3, 46.60, 46.56, 41.9, 40.8, 40.4, 31.1, 29.7, 25.8, 25.7; HRMS (FAB) calculated for $C_{12}H_{13}NO_4$ $[M + Na]^+$ 258.0742 found 258.0750.

Polymerization of **4** to give **6**

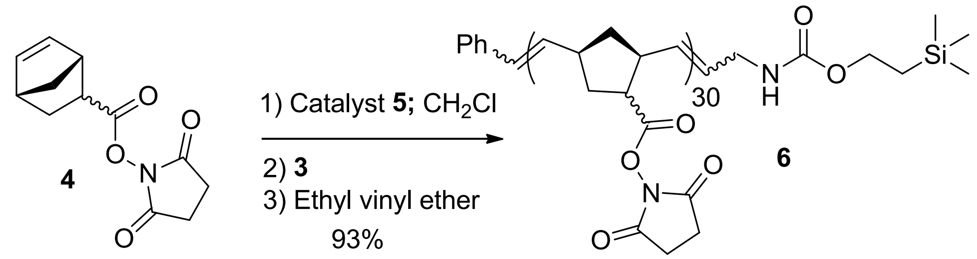

To a solution of **4** (0.48 g, 2.05 mmol) in 10 mL dry CH_2Cl_2 in a capped vial at −78 °C was added a solution of catalyst **5** [5] (0.06 g, 0.07 mmol) in 5 mL dry CH_2Cl_2 quickly via a syringe. The reaction was allowed to warm to room temperature upon which the solution turned from bright green to brown after about 2 minutes. After 40 minutes a solution of **3** (0.13 g, 0.34 mmol) in 5 mL dry CH_2Cl_2 was added to the reaction via a syringe. After 20 min 0.5 mL of ethyl vinyl ether (0.38 g, 5.22 mmol) was added via a syringe, and the reaction solution was transferred using a Pasteur pipette to 4 different test tubes each containing about 10 mL of ether, forming a light brown precipitate. The test tubes were centrifuged and the ether decanted, and the precipitate was washed by adding 10 mL of ether into the test tubes and agitating the mixture with a Pasture pipette then centrifuging and decanting the ether. Washing was repeated until the ether was clear. The grey precipitate was collected and dried under vacuum to give **6** (0.44 g, 0.06 mmol) as a grey solid 93%. Partial ^1H NMR (400 MHz, d_6-acetone) δ 7.24–7.21 (m, 5H, **H-Ph**) 5.8–5.4 (m, 84H, **H-olefinic**) 0.0 (s, 9H, **H-TMS**).

Preparation of **7**

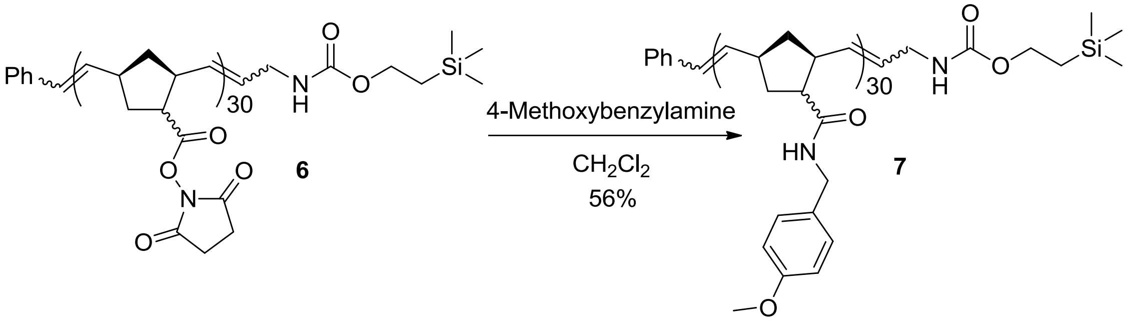

To a solution of **6** (0.09 g, 0.013 mmol) in 5 mL dry CH_2Cl_2 was added 250 µL of 4-methoxybenzyl amine (0.26 g, 0.93 mmol), and the mixture was stirred for 24 h. The solvent was evaporated, the residue was dissolved in 20 mL THF and filtered through a plug of cotton, and then the cotton was washed with 10 mL THF. The filtrate was then transferred to a dialysis tube and dialyzed in 200 mL THF reservoir (12 h × 3). The solution in the tube was collected, and the excess solvent was evaporated and dried to give **7** (0.06 g) 56% as a white film partial ^{1}H NMR (400 MHz, $CDCl_3$) δ 7.13 (bs, 77H, **H-aromatic**) 6.80 (bs, 77H, **H-aromatic**) 5.28–5.17 (m, 77H, **H-olefinic**) 3.74–3.72 (m, 120H, **H-OMe**) 0.02 (s, 9H, **H-TMS**) MW 6372, PDI 1.38 (MALDI-ToF).

1-Amino -11- azido-3,6,9-trioxaundecane (**8**)

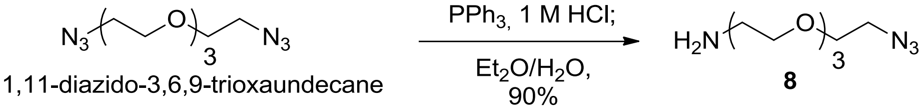

60 mL of 1M HCl was added to a flask containing 1,11-diazido-3,6,9-trioxaundecane [6] (4 g, 16.4 mmol) at 0 °C. While vigorously stirring the mixture a solution of PPh3 (5 g, 19.1 mmol) in 60 mL Et_2O was added dropwise to the flask using an addition funnel then 10 mL of Et2O as used to rinse the funnel. The mixture was allowed to warm to room

temperature. After 24 h the white precipitate was filtered and the aqueous layer washed with 50 mL Et_2O three times. The aqueous layer was cooled at 0 °C and 30 g of KOH was added. After the effervescence stopped the mixture was extracted in DCM (20 mL × 5). The CH_2Cl_2 layers were combined and dried over Na_2SO_4. Excess solvent was evaporated, and the product was dried under vacuum to give **8** as pale yellow oil 90% (3.23 g, 14.7 mmol). ^1H NMR (300 MHz, $CDCl_3$) δ 3.70–3.51 (m, 10H) 3.48 (t, J = 5.2 Hz, 2H) 3.33 (t, J = 5.0 Hz, 2H) 2.83 (t, J = 5.1 Hz, 2H) 2.78 (s, 2H); ^{13}C NMR (75 MHz, $CDCl_3$) δ 72.7, 70.7, 70.2, 70.0, 50.7, 41.5. HRMS (FAB) calculated for $C_8H_{18}N_4O_3$ $[M + Na]^+$ 241.1277 found 214.1272.

Azido-polymer **9**

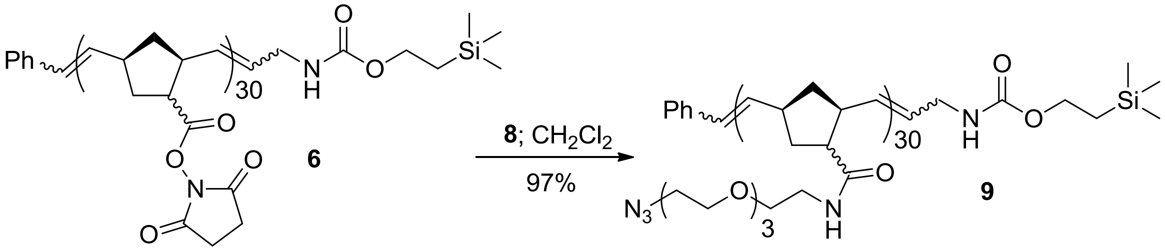

To a solution of **6** (0.4 g, 0.06 mmol) in 5 mL dry CH_2Cl_2 was added a solution of **8** (0.7 g, 3.4 mmol) in 5 mL dry CH_2Cl_2, and the mixture was stirred for 24 h. The solvent was evaporated, and the brown residue was dissolved in 10 mL MeOH, transferred into a dialysis tube and dialyzed in 200 mL MeOH reservoir (12 h x 3). The solution in the dialysis tube was collected, evaporated and dried under vacuum to give **9** as a light brown sticky gel 97% (0.6 g, 0.06 mmol). Partial ^1H NMR (300 MHz, $CDCl_3$) δ 5.55–5.02 (m, 75H, **H-olefinic**) 0.00 (s, 9H, **H-TMS**).

Galactosyl glycopolymer **11a**

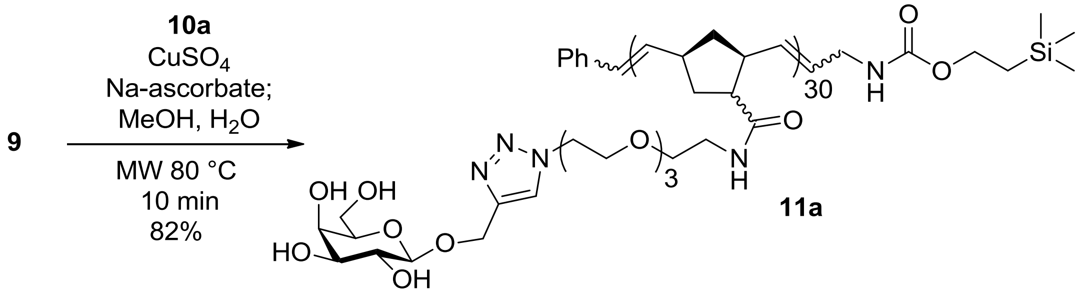

Solutions of poly-azide **9** (0.14 g, 0.013 mmol) in 2 mL MeOH, propargyl galactoside **10a** [7] (0.2 g, 0.92 mmol) in 0.5 mL H_2O, $CuSO_4$•$5H_2O$ (8 mg, 0.03 mmol) in 0.5 mL H_2O, Na-ascorbate (12 mg, 0.06 mmol) in 0.5 mL were added to a 2–5 mL microwave vial charged with a stir bar in that order. The reaction was run in a microwave at 80 °C for 10 min. The resulting solution was transferred into a vial containing 1 g CupriSorb®[8] and 5 mL H_2O, stirred overnight to remove copper ions, and filtered through a plug of cotton. The CupriSorb® [8] was washed with 10 mL H_2O and the collected filtrate dialyzed against 200 mL H_2O (12 h x 4). The solution was then concentrated to about 2 mL by centrifuge filtration. The H_2O was then azeotroped with 1 mL toluene and evaporated on a rotovap at 40 °C (x 3). The product was dried under vacuum to give glycopolymer **11a** (0.2 g, 0.012 mmol) as a brown film 82%. Partial ^{1}H NMR (400 MHz, D_2O) δ 8.15 (s, 40H, **H-triazole**) 5.38 (bs, 80H, **H-olefinic**) 5.03 (d, J = 12.4 Hz, 40H, **H-Gal**) 4.88 (d, J = 11.7, 40H, **H-Gal**) 4.65 (bs, 80H, **H-Gal**) 4.51 (d, J = 7.7 Hz, 40H, **H-Gal**) 0.04 (s, 9H, **H-TMS**).

Mannosyl glycopolymer **11b**

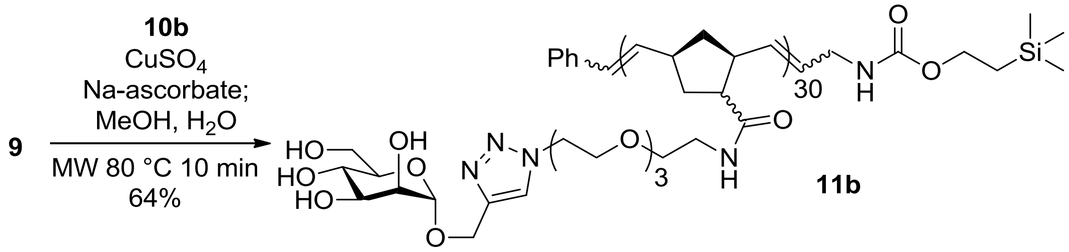

Mannosyl glycopolymer **11b** was obtained using the same procedure reported for galactose glycopolymer **11a**. 64% of **11b** was recovered. Partial ^{1}H NMR (300 MHz, D_2O) δ 8.14 (s, 37H, **H-triazole**) 5.37 (bs, 74H, **H-olefinic**) 4.98 (s, 40H, **H-Man**) 0.02 (s, 9H, **H-TMS**).

3-sulfogalactose glycopolymer **11c**

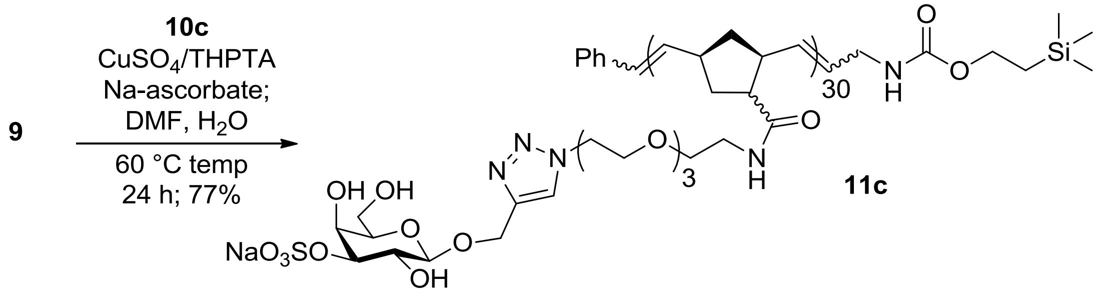

Solutions of poly-azide **9** (0.15 g, 0.014 mmol) in 2 mL DMF, propargyl 3' sulfogalactoside **10c** [7] (0.20 g, 0.625 mmol) in 0.5 mL H_2O, $CuSO_4$•$5H_2O$ (8 mg, 0.03 mmol) and THPTA [8] (65 mg, 0.15 mmol) mixture in 0.5 mL H_2O, Na-ascorbate (12 mg, 0.06 mmol) in 0.5 mL H_2O were added to a vial charged with a stir bar in that order. The reaction was run at 60 °C for 24 h. 10 mL H_2O was added and the solution transferred into a vial containing 1 g CupriSorb® [8], stirred overnight to remove copper ions and filtered through a plug of cotton. The CupriSorb® [8] was washed with 10 mL H_2O and the collected filtrate dialyzed against 200 mL H_2O (12 h x 4). The solution was

then concentrated to about 2 mL by centrifuge filtration and the H_2O was then azeotroped with 1 mL toluene and evaporated on a rotovap at 40 °C (x 3) followed by 1 mL heptane. The product was dried under vacuum to give glycopolymer **11c** (0.23 g, 0.011 mmol) as a light brown film 77%. ^{1}H NMR (400 MHz, D_2O) δ 8.17 (s, 40H, **H-triazole**) 5.40 (bs, 80H, **H-olefinic**) 5.05 (d, *J* = 12.6 Hz, 40H, **H-SGal**) 4.91 (d, *J* = 11.9, 40H, **H-SGal**) 4.65 (bs, 80H, **H-SGal**) 4.35 (d, *J* = 7.6 Hz, 40H, **H-SGal**) 0.06 (s, 9H, **H-TMS**).

Amino-terminated galactosylated glycopolymer **12a**

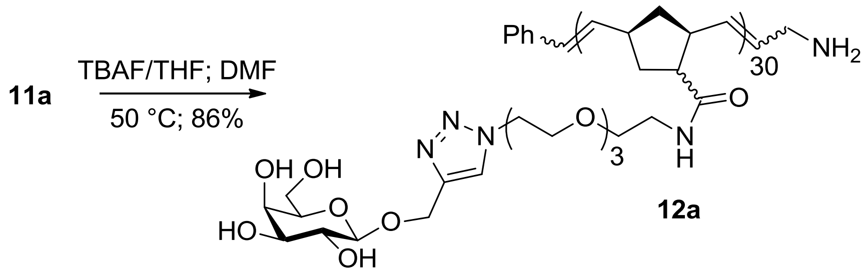

To a solution of glycopolymer **11a** (0.12 g, 7 µmol) in 2 mL dry DMF was added 1 mL of 1 M TBAF solution in THF and the mixture stirred for 24 h at 50 °C. 1 mL of 1% NaCl solution and 10 mL H_2O was added and the mixture dialyzed against 200 mL H_2O (12 h x 4). The solution was then concentrated to about 2 mL by centrifuge filtration and the H_2O was then azeotroped with 1 mL toluene then evaporated on a rotovap at 40 °C (x 3) followed by 1 mL heptane. The product was dried under vacuum to give glycopolymer **12a** (0.10 g, 6 µmol) as a light brown film 86%. ^{1}H NMR (400 MHz, D_2O) δ 8.15 (s, 40H, **H-triazole**) 5.37 (bs, 80H, **H-olefinic**) 5.03 (d, *J* = 12.6 Hz, 40H, **H-Gal**) 4.93-4.83 (m, 40H, **H-Gal**) 4.65 (bs, 80H, **H-Gal**) 4.51 (d, *J* = 7.7 Hz, 40H, **H-Gal**).

Amino-terminated mannosylated glycopolymer **12b**

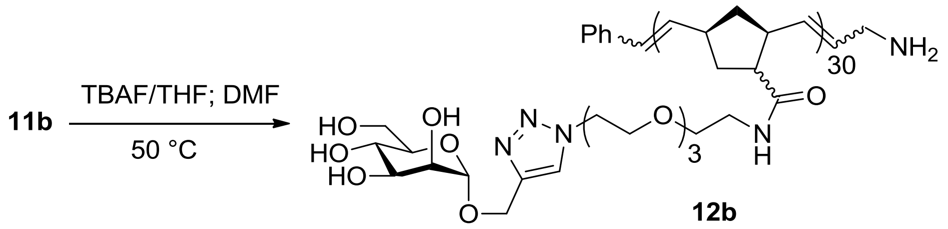

Amino-terminated mannosylated glycopolymer **12b** was obtained using the same procedure reported for amino-terminated galactose glycopolymer **12a**. 55% of polymer **12b** was recovered. Partial ^{1}H NMR (300 MHz, D_2O) δ 8.13 (s, 37H, **H-triazole**) 5.37 (bs, 77H, **H-olefinic**) 4.98 (s, 39H, **H-Man**).

Amino-terminated 3'-sulfogalactosylated glycopolymer **12c**

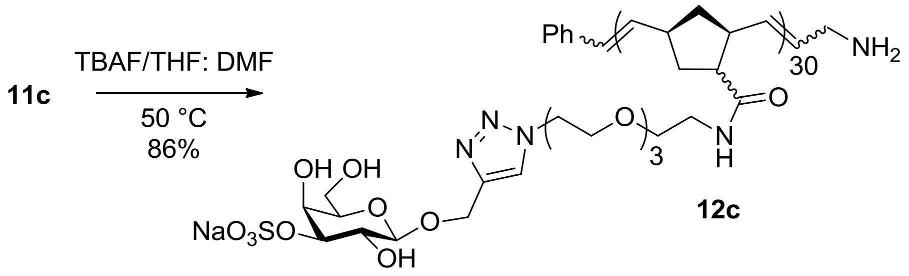

To a solution of glycopolymer **11c** (0.15 g, 8 μmol) in 2 mL dry DMF was added 1 mL of 1 M TBAF solution in THF, and the mixture was stirred for 24 h at 50 °C. 10 mL H_2O was added, and the mixture was dialyzed against 200 mL H_2O (12h x 4). The solution was then subjected to Na^+ exchange chromatography (Na^+ form generated from Amberlite IR-I20 resin H^+ form using brine) to remove TBA cation and concentrated to about 2 mL by centrifuge filtration, and the H_2O was then azeotroped with 1 mL toluene then evaporated on a rotovap at 40 °C (x 3) followed by 1 mL heptane. The product was then dried under vacuum to give glycopolymer **12c** (0.13 g, 7 μmol) as a light brown film

86%. ^{1}H NMR (400 MHz, D_2O) δ 8.16 (s, 40H, **H-triazole**) 5.46 (bs, 80H, **H-olefinic**) 5.05 (d, *J* = 12.6 Hz, 40H, **H-SGal**) 4.71–4.58 (m, 40H, **H-SGal**) 4.51 (d, *J* = 7.6 Hz, 40H, **H-SGal**).

N-(2-(2-(2-(2-isothiocyanatoethoxy)ethoxy)ethoxy)ethyl)-4-(pyren-2-yl)butanamide **15**

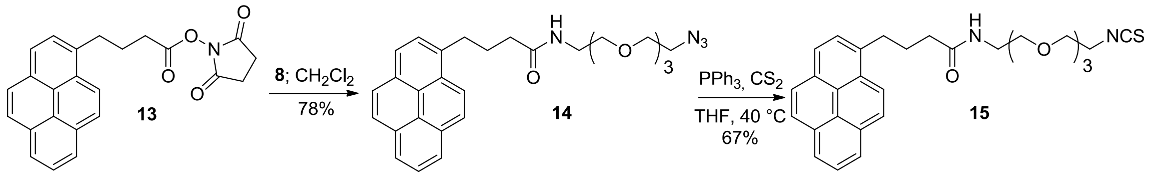

To a solution of 13 [8] (0.15 g, 0.4 mmol) in dry CH_2Cl_2 was added a solution of **8** (0.13 g, 0.6 mmol) in dry CH_2Cl_2, and the mixture was stirred until TLC (CH_2Cl_2) showed that **13** had been completely consumed (ca. 3 h). The solvent was then evaporated and the brown residue purified by automatic flash chromatography (100:0 to 95:5 CH_2Cl_2:MeOH). The solvent was evaporated and product dried under vacuum to give **14** (0.15 g, 0.31 mmol) 78% as a yellow oil. To a solution of **14** (0.1 g, 0.2 mmol) in 10 mL THF:CS_2 1:1 mixture was added 0.08 g of polymer bound PPh_3 (3 mmol/g of polymer = 0.065 g, 0.25 mmol of PPh_3), and the reaction mixture was heated at 40 °C for 24 h. The mixture was then filtered, the solvent evaporated, and the product dried under vacuum to give **15** (0.07 g, 0.14 mmol) 67% as a yellow oil. ^{1}H NMR (300 MHz, $CDCl_3$) δ 8.32–7.85 (m, 9H) 6.00 (bs, 1H) 3.67–3.46 (m, 16H) 3.39 (t, *J* = 7.43 Hz, 2H) 2.33–2.17 (m, 4H); ^{13}C NMR (75 MHz, $CDCl_3$) δ 172.8, 136.1, 131.5, 131.0, 130.0, 128.9, 127.6, 127.50, 127.47, 126.8, 126.0, 125.2, 124.90, 124.87, 123.6, 70.8, 70.7, 70.5, 70.3, 69.9, 69.2, 45.2, 39.3, 36.1, 32.9, 27.5 HRMS (FAB) calculated for $C_{29}H_{32}N_2O_4S$ [M + Na]$^+$ 527.1981 found 527.1966.

Pyrene-end-labeled galactosyl glycopolymer **16a**

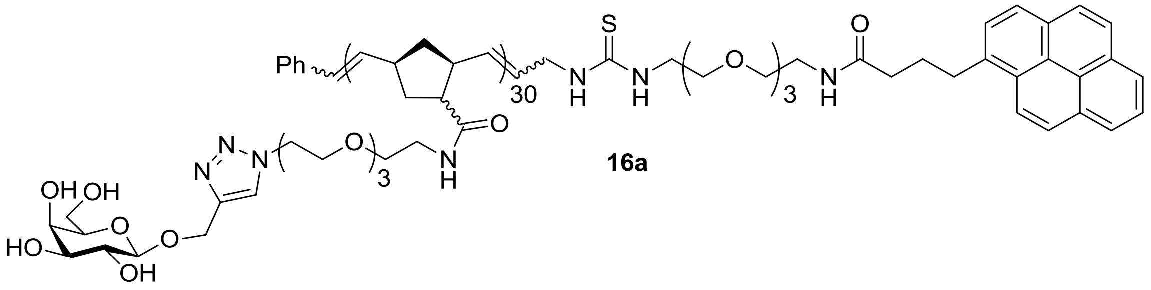

16a

To a solution of galactosyl glycopolymer **12a** (80 mg, 5 μM) in 2 mL DMF was added 527 μL of 9.5 M **15** (9.6 mg, 19 μM) in DMF, and the mixture was stirred at 50 °C overnight. The resulting solution was dialyzed from 200 mL DMF for 4 h to remove excess **15** then dialyzed from 200 mL MeOH:H2O 1:1 mixture (12 h x 3) and concentrated to about 2 mL by centrifuge filtration, and the H_2O was then azeotroped with 1 mL toluene and evaporated on a rotovap at 40 °C (x 3) followed by 1 mL heptane. The product was then dried under vacuum to give glycopolymer **16a** (0.07 g, 4 μmol) as a light brown film, 81% recovered.

Pyrene-end-labeled mannosyl glycopolymer **16b**

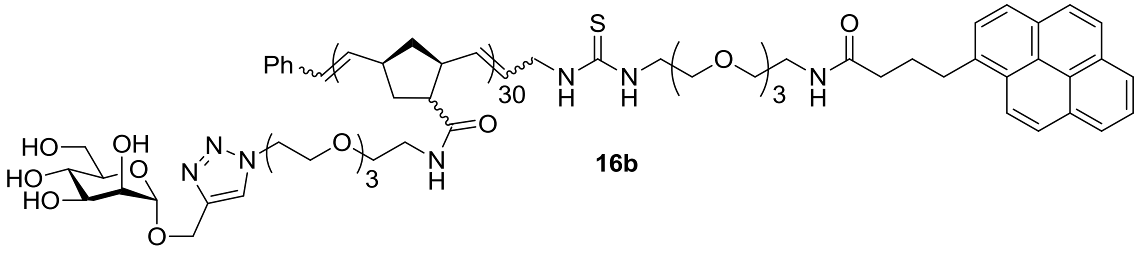

16b

Pyrene-end-labeled mannose glycopolymer **16b** was obtained using the same procedure reported for pyrene-end-labeled galactose glycopolymer **16a**. 96% of **16b** was recovered.

Pyrene-end-labeled 3'-sulfogalactosyl glycopolymer **16c**

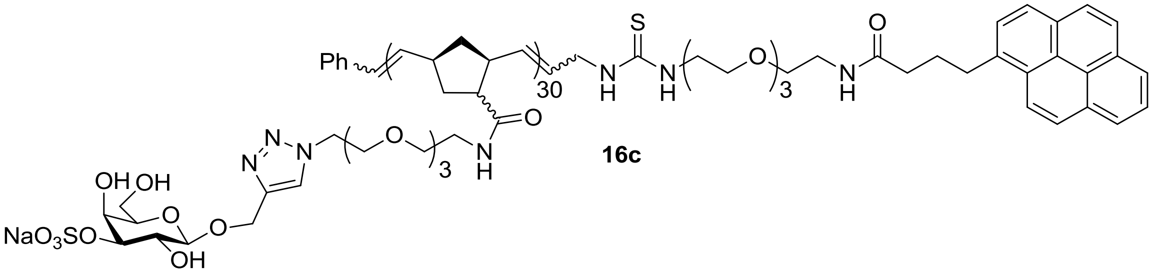

16c

Pyrene-end-labeled 3'-sulfogalactosyl glycopolymer **16c** was obtained using the same procedure reported for pyrene-end-labeled galactose glycopolymer **16a**. 65% of **16c** was recovered.

2. References

1. Pangborn, A. B.; Giardello, M. A.; Grubbs, R. H.; Rosen, R. K.; Timmers, F. J. Safe and convenient procedure for solvent purification. *Organometallics* **1996**, *15,* 1518–1520.
2. Rosowsky, A.; Wright, J. E. N ω.-alkoxycarbonylation of α,ω-diamino acids with 2-(trimethylsilyl)ethyl 4-nitrophenyl carbonate. *J. Org. Chem*. **1983**, *48,* 1539–1541.
3. He, Z.; Nadkarni, D. V.; Sayre, L. M.; Greenaway, F. T. Mechanism-based inactivation of porcine kidney diamine oxidase by 1,4-diamino-2-butene. *Biochim. et Biophys. Acta,* **1995**, 1235, 117–127.
4. Strong, L. E.; and Kiessling, L. L. A general synthetic route to defined, biologically active multivalent arrays. *J. Am. Chem. Soc.* **1999**, *121,* 6193–6196.
5. Love, J. A.; Morgan, J. P.; Trnka, T. M.; Grubbs, R. H. A practical and highly active ruthenium-based catalyst that effects the cross metathesis of acrylonitrile. *Angew. Chem. Int. Ed.* **2002**, *41,* 4035–4037.
6. Schwabacher, A. W.; Lane, J. W.; Schiesher, M. W.; Leigh, K. M.; Johnson, C. W. Desymmetrization reactions: Efficient preparation of unsymmetrically substituted linker molecules. *J. Org. Chem*. **1998**, *63,* 1727–1729.
7. Zhao, J.; Liu, Y.; Park, H.; Boggs, J. M.; Basu, A. Carbohydrate-coated fluorescent silica nanoparticles as probes for the galactose/3-sulfogalactose carbohydrate−carbohydrate interaction using model systems and cellular binding studies. *Bioconjugate Chem*. **2012**, *23,* 1166–1173.
8. Hong, V.; Presolski, S. I.; Ma, C.; Finn, M. G. Analysis and optimization of copper-catalyzed azide-alkyne cycloaddition for bioconjugation. *Angew. Chem. Int. Ed.* **2009**, *48,* 9879–9883.
9. Wu, Z. Synthesis and characterization of active ester-functionalized fluorescent polymers: New materials for protein conjugation. *J. Appl. Polym. Sci.* **2008**, *110,* 777–783.

3. MALDI–ToF analysis

MALDI–ToF samples were prepared as follows; Polymer **7** (ca. 30 mg) was dissolved in 400 µL of THF. 70 µL water containing 0.1% TFA and 30 µL acetonitrile were added to about 6 mg of super DHB, and the mixture was vortexed to provide a clear solution. 10 µL of the polymer solution and 10 µL of the matrix solution were mixed, and then 1 µL of the mixture was applied directly to the MALDI target plate and allowed to dry completely before acquisition.

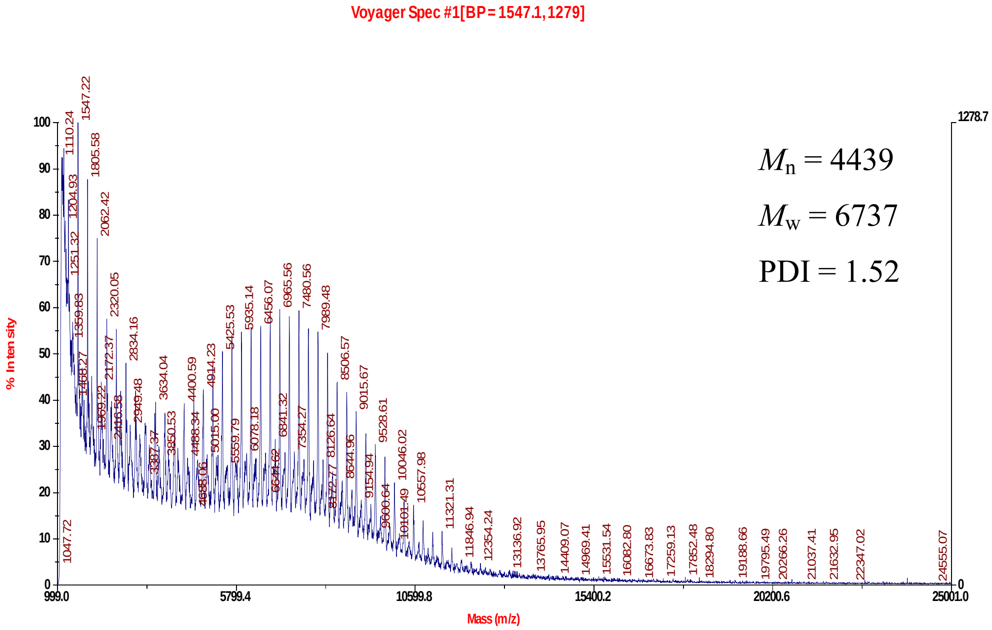

Figure S1: MALDI spectrum of polymer **7**.

4. GPC analysis

GPC samples were prepared as follows; 10 µL of 0.2% **6** (0.004 g in 2 mL THF) was injected and eluted using HPLC-grade THF flowing at 1 mL/min at room temperature; Absorbance was monitored at 254 nm. GPC molecular weight (MW) was obtained from a polystyrene standards calibration curve. M_w = 7469; M_n = 4791; DPI = 1.56.

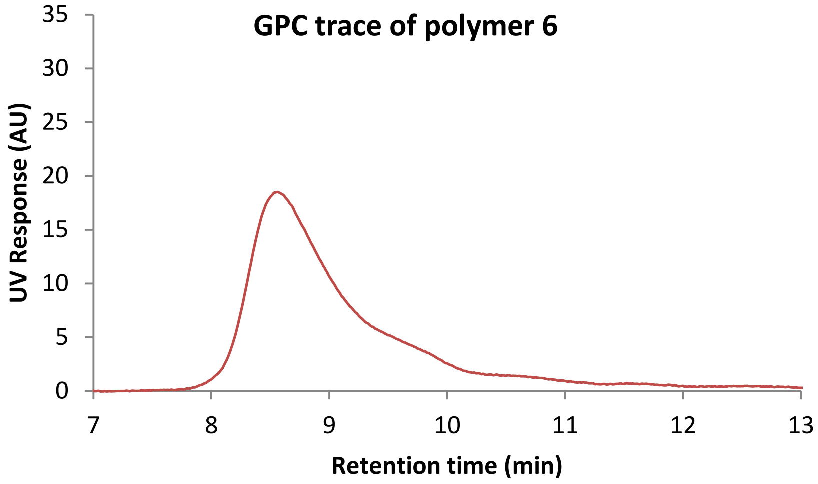

Figure S2: GPC chromatogram of polymer **6**.

5. Capping efficiency table

Table S1

n[b] \ Time[a]	5 min	20 min	1 h	15 h
	**Ph:TMS**[c]	**Ph:TMS**[c]	**Ph:TMS**[c]	**Ph:TMS**[c]
15	5:7 (1)	5:8.3 ± 1.2 (3)	5:8.7 ± 0.6 (3)	5:9 (1)
30	5:6 (1)	5:9.4 ± 0.8 (11)	5:9.7 ± 0.6 (3)	5:9 (1)
50[d]	5:4	5:11.7 ± 0.6 (3)	5:11 ± 1 (3)	5:11 (1)

[a] Reaction time after addition of a solution of terminating agent **3** to the reaction mixture before quenching with ethyl vinyl ether.
[b] Theoretical degree of polymerization determined by the molar ratios of monomer **4** to catalyst **5** used.
[c] The integral for the aromatic protons (derived from the Ph in catalyst **5**) was set to 5. The values are the ratios of the integral for aromatic protons to the TMS protons in the ^{1}H NMR spectra of polymers **6**. The number in parentheses refers to the number of times the polymerization and capping reactions were carried out.
[d] Although the TMS integrations for the 50mer are greater than 9 at 20 minutes and beyond, the degree of polymerization corresponding to this integration is also high, at approximately 80. However, if these spectra are reintegrated by setting the integral for the TMS peak to 9, the degree of polymerization is found to be 64. This is in accordance with the M_w determined by GPC - 6693, which corresponds to a degree of polymerization of 63. Thus, we believe that the higher TMS integrations are a result of the error associated with integration of the small and broad aromatic peaks in the spectra of the 50mers. Moreover, the lack of a significant change in the integration values from 20 min to 15 hours indicates that secondary metathesis is unlikely to be the cause of the higher TMS integrations.

6. IR Spectra of Polymers

IR spectra were obtained from a dried film formed from a methanolic solution of polymer on NaCl discs.

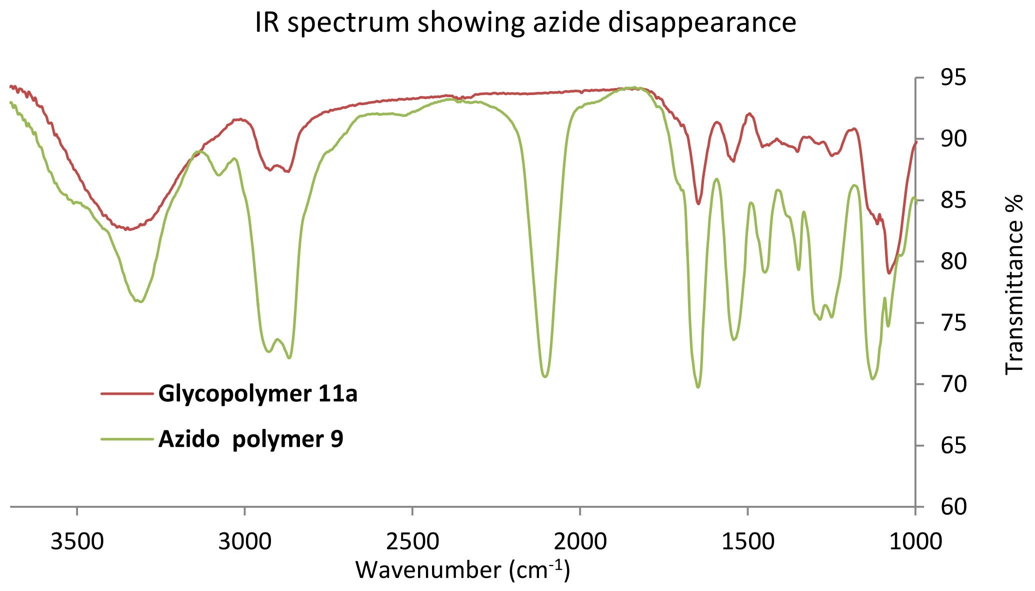

Figure S3: Overlay IR spectra of azido polymer **9** and glycopolymer **11a**.

7. Determination of extent of pyrene end-labeling

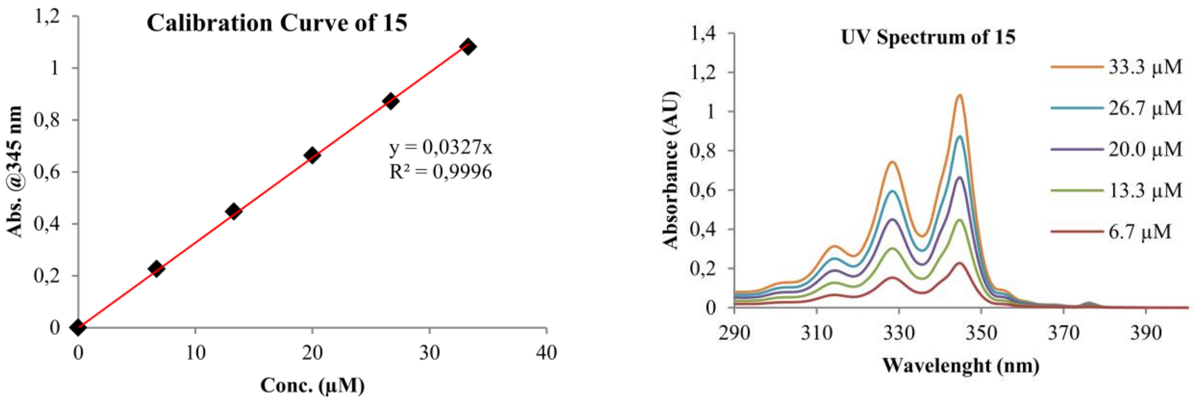

Figure S4: UV spectrum and calibration curve of **15**.

Molar extinction coefficient Ɛ of **15** = 32,700 L mol^{-1} cm^{-1}.

Polymer 16a

25 mg of pyrene labeled galactose glycopolymer **16a** was dissolved in 1 mL DMF to make 1.44 mM stock solution.

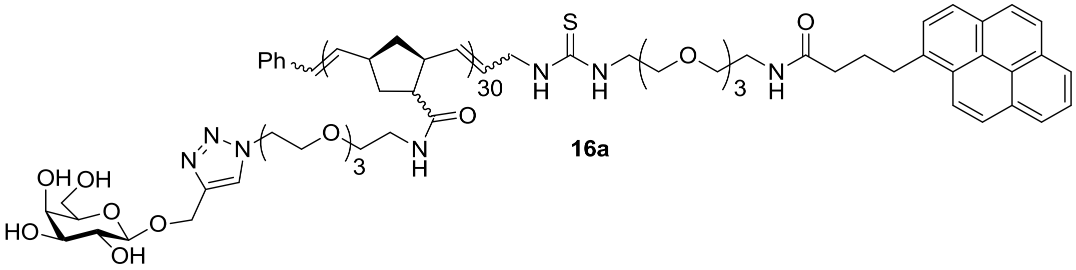

16a

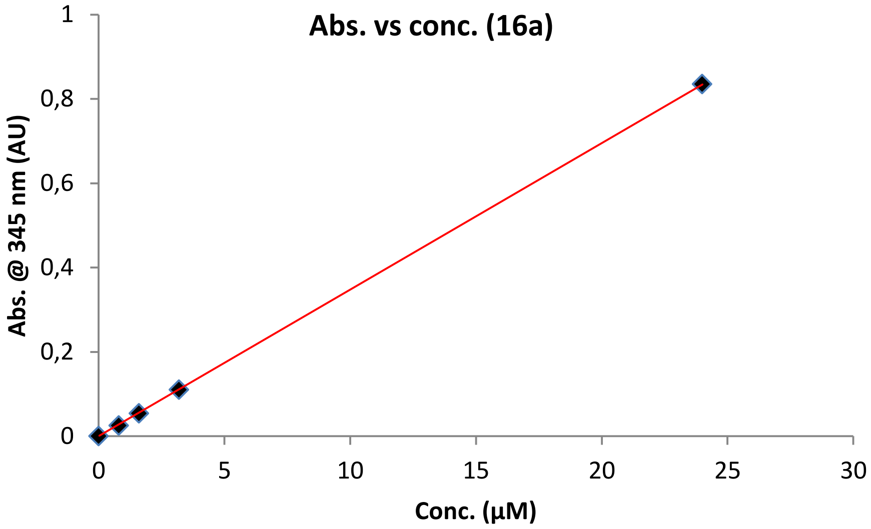

Figure S5: Graph of absorbance @ 345 nm against concentration for polymer **16a**.

Table S2: Percent pyrene labeling of polymer **16a**.

**16a** Conc. (µM)	Abs. @ 345 nm	**PY µmol/g**	**% label**
0.8	0.0251	**55**	**96**
1.6	0.0537	**59**	**103**
3.2	0.1099	**61**	**105**
24	0.8343	**61**	**106**
Average		**59**	**103**

Polymer 16b

26 mg of pyrene labeled mannose glycopolymer **16b** dissolved in 1 mL DMF to make 1.5 mM stock solution.

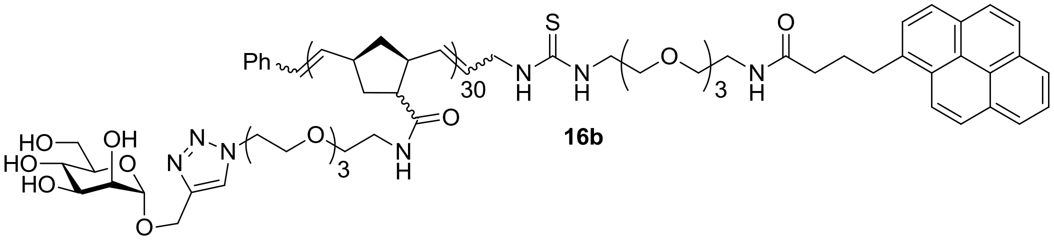

16b

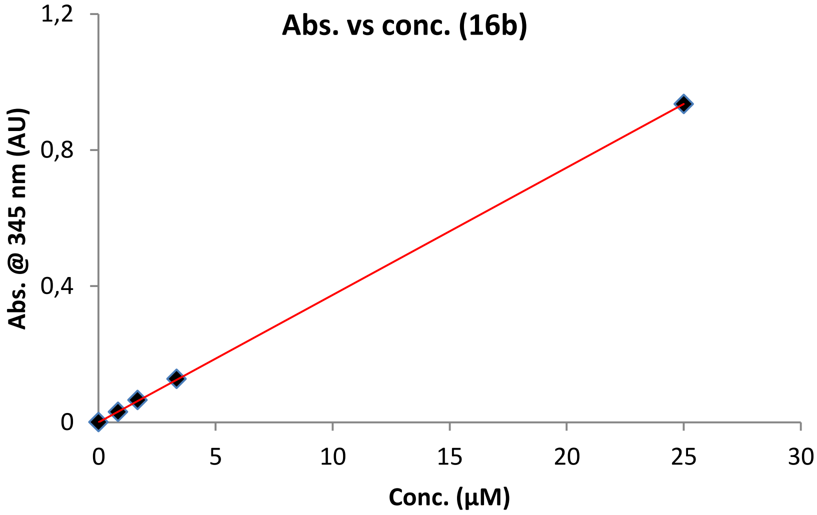

Figure S6: Graph of absorbance @ 345 nm against concentration for polymer **16b**.

Table S3: Percent pyrene labeling of polymer **16b**.

**16b** Conc. (µM)	Abs. @ 345 nm	**PY µmol/g**	**% Label**
0.83	0.0307	**65**	**113**
1.67	0.0648	**68**	**118**
3.33	0.1272	**67**	**116**
25	0.9350	**66**	**114**
Average		**68**	**116**

Polymer 16c

24 mg of pyrene labeled 3'-sulfogalactose glycopolymer **16c** dissolved in 1mL DMF to make 1.18 mM stock solution.

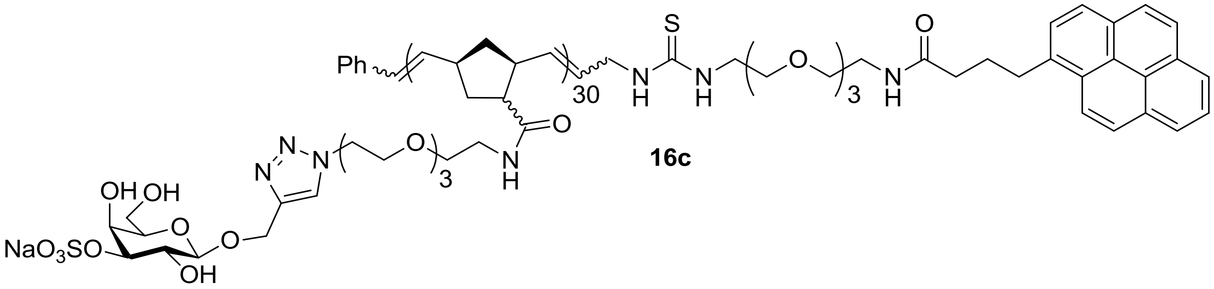

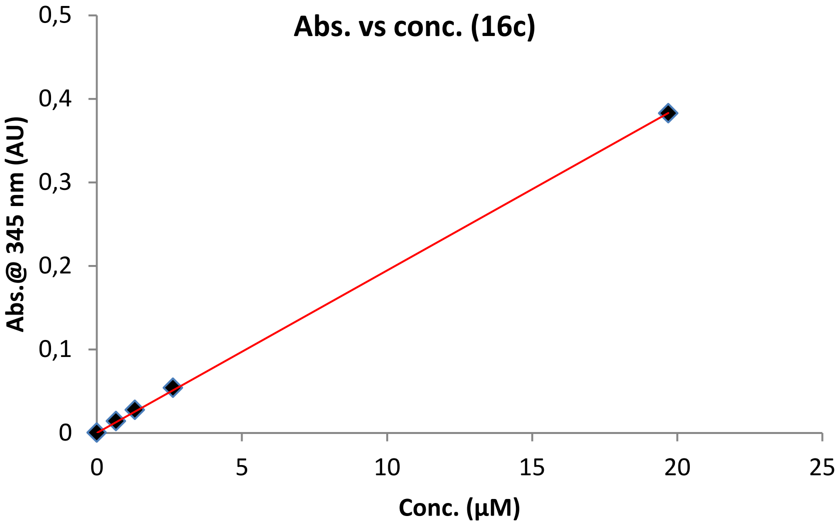

Figure S7: Graph of absorbance @ 345 nm against concentration for polymer **16c**.

Table S4: Percent pyrene labeling of polymer **16c**.

**16c** Conc. (µM)	Abs. @ 345 nm	**PY µmol/g**	**% Label**
0.66	0.0140	**32**	**65**
1.31	0.0275	**32**	**65**
2.63	0.0538	**31**	**63**
19.7	0.3826	**29**	**59**
Average		**31**	**63**

8. Teledyne ISCO COMBI flash R_f chromatograms

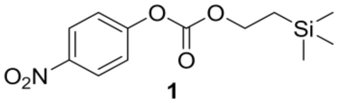

1

RediSep Column: Silica 40g Gold
Flow Rate: 40 ml/min
Equilibration Volume: 5.0 CV
Initial Waste: 0.0 CV
Air Purge: 1.0 min
Solvent A: A1 hexane
Solvent B: B1 ethyl acetate

Peak Tube Volume: Max.
Non-Peak Tube Volume: Max.
Loading Type: Solid
Wavelength 1 (red): 254nm
Peak Width: 2 min
Threshold: 0.20 AU
All Wavelength (orange): 200nm - 360nm
Peak Width: 2 min
Threshold: 0.20 AU

Run Notes:

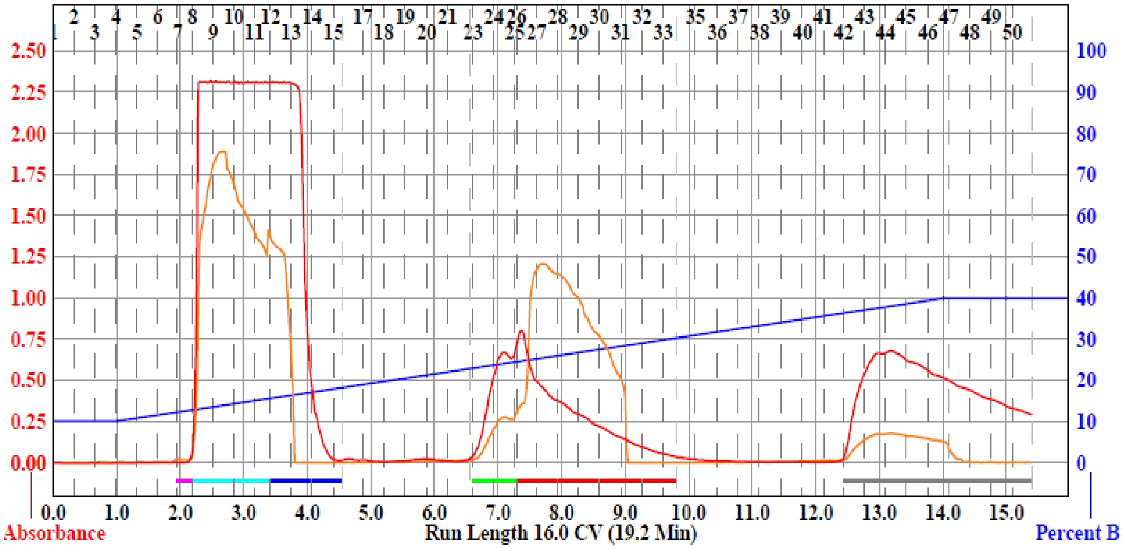

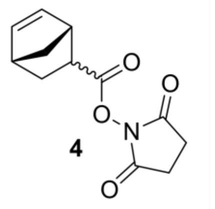

4

RediSep Column: Silica 24g Gold
Flow Rate: 35 ml/min
Equilibration Volume: 5.0 CV
Initial Waste: 0.0 CV
Air Purge: 1.0 min
Solvent A: A2 dichloromethane
Solvent B: B1 ethyl acetate

Peak Tube Volume: Max.
Non-Peak Tube Volume: Max.
Loading Type: Solid
Wavelength 1 (red): 254nm
Peak Width: 1 min
Threshold: 0.20 AU
All Wavelength (orange): 200nm - 360nm
Peak Width: 1 min
Threshold: 0.20 AU

Run Notes:

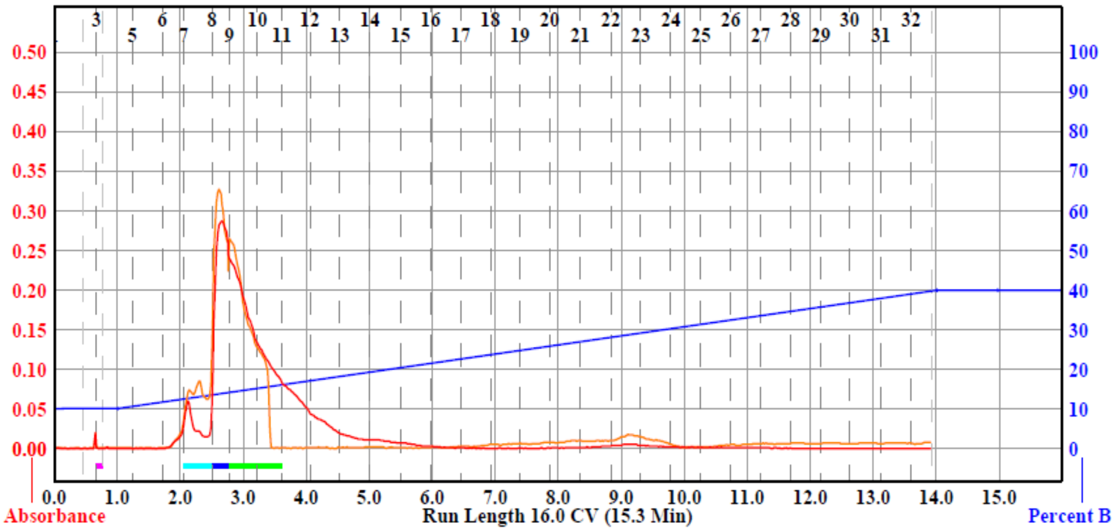

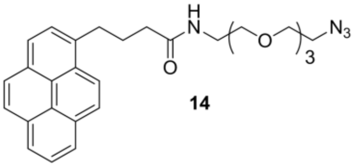

14

RediSep Column: Silica 24g Gold
SN: E0410597DC182 Lot: 2115201090W
Flow Rate: 35 ml/min
Equilibration Volume: 5.0 CV
Initial Waste: 0.0 CV
Air Purge: 1.0 min
Solvent: A2 methanol
Solvent: B2 dichloromethane

Peak Tube Volume: Max.
Non-Peak Tube Volume: Max.
Loading Type: Solid
Wavelength 1 (red): 254nm
Peak Width: 1 min
Threshold: 0.20 AU
Wavelength 2 (purple): 280nm

Run Notes:

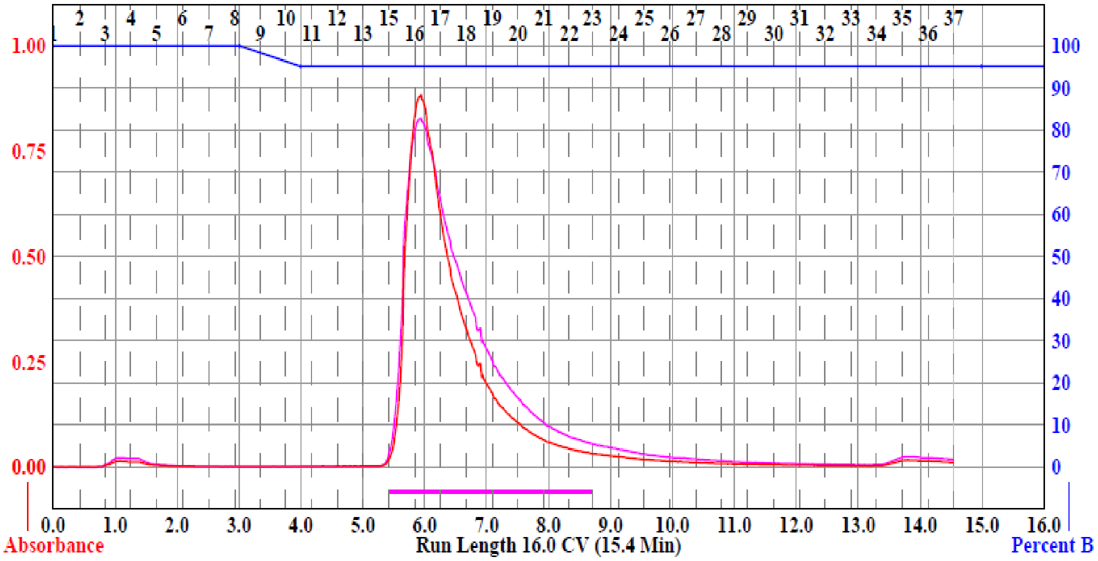

9. NMR Spectra

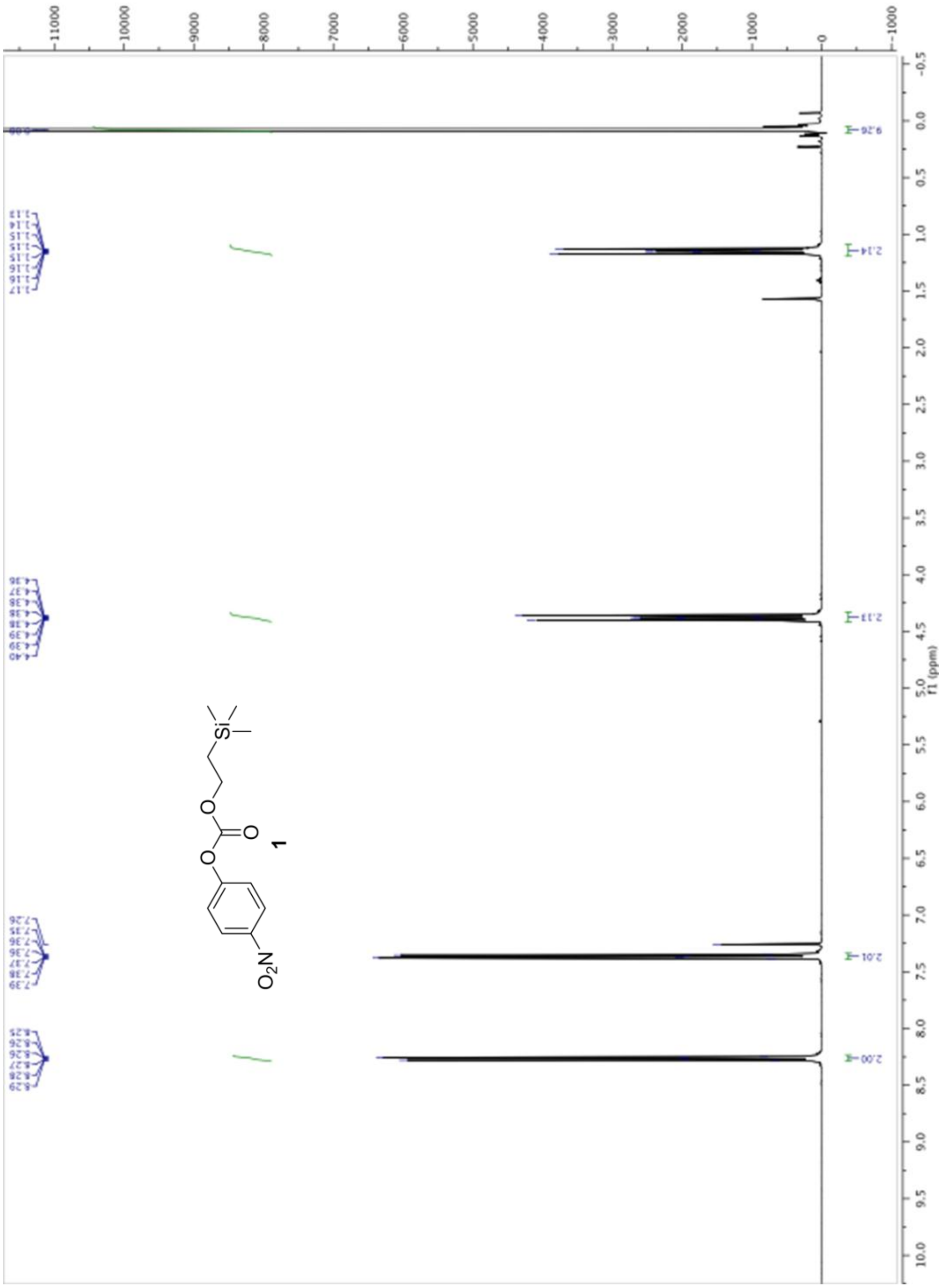

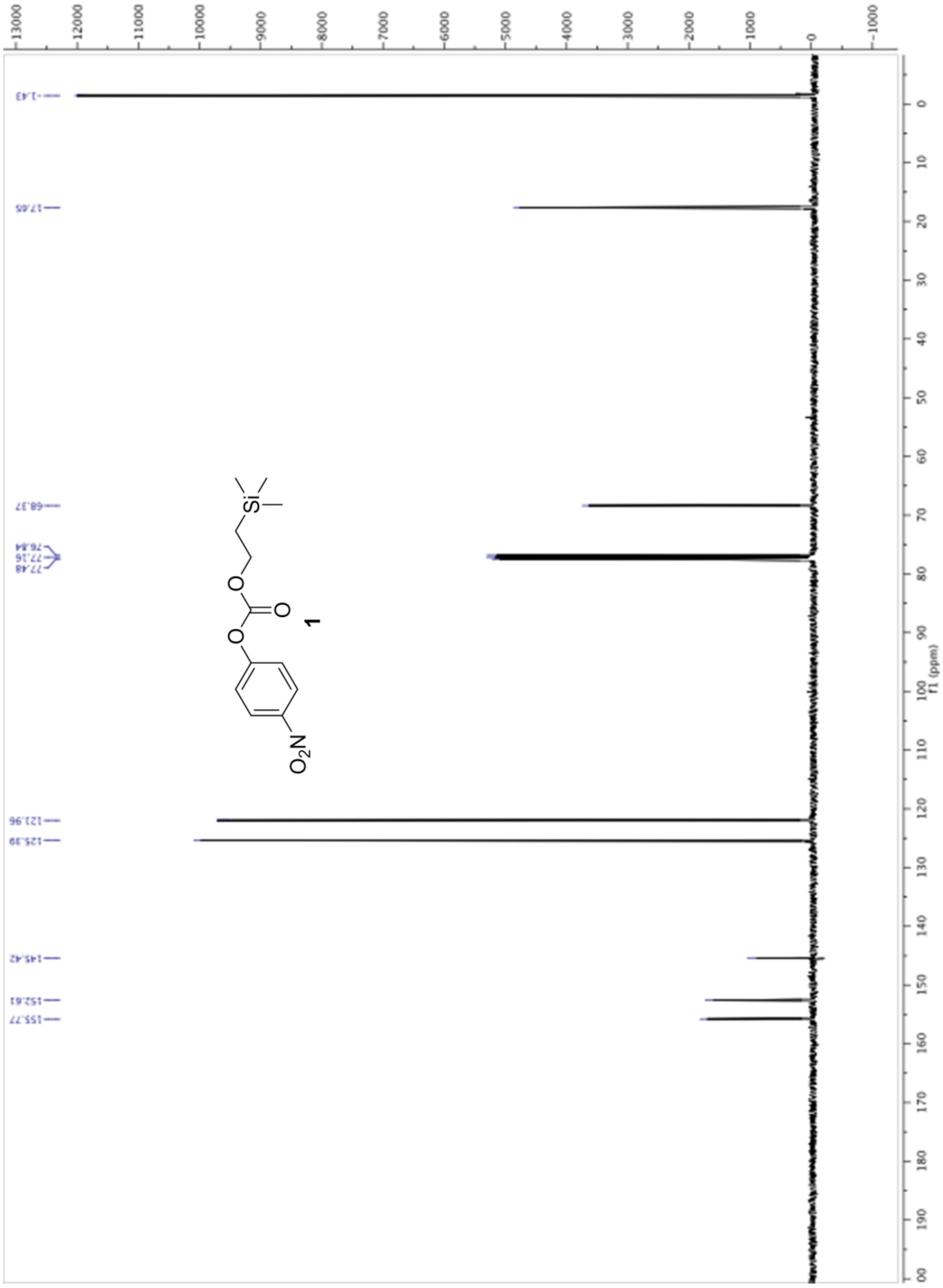
-1.43
17.65
68.37
76.84
77.16
77.48
121.96
125.39
145.42
152.61
155.77
f1 (ppm)
1

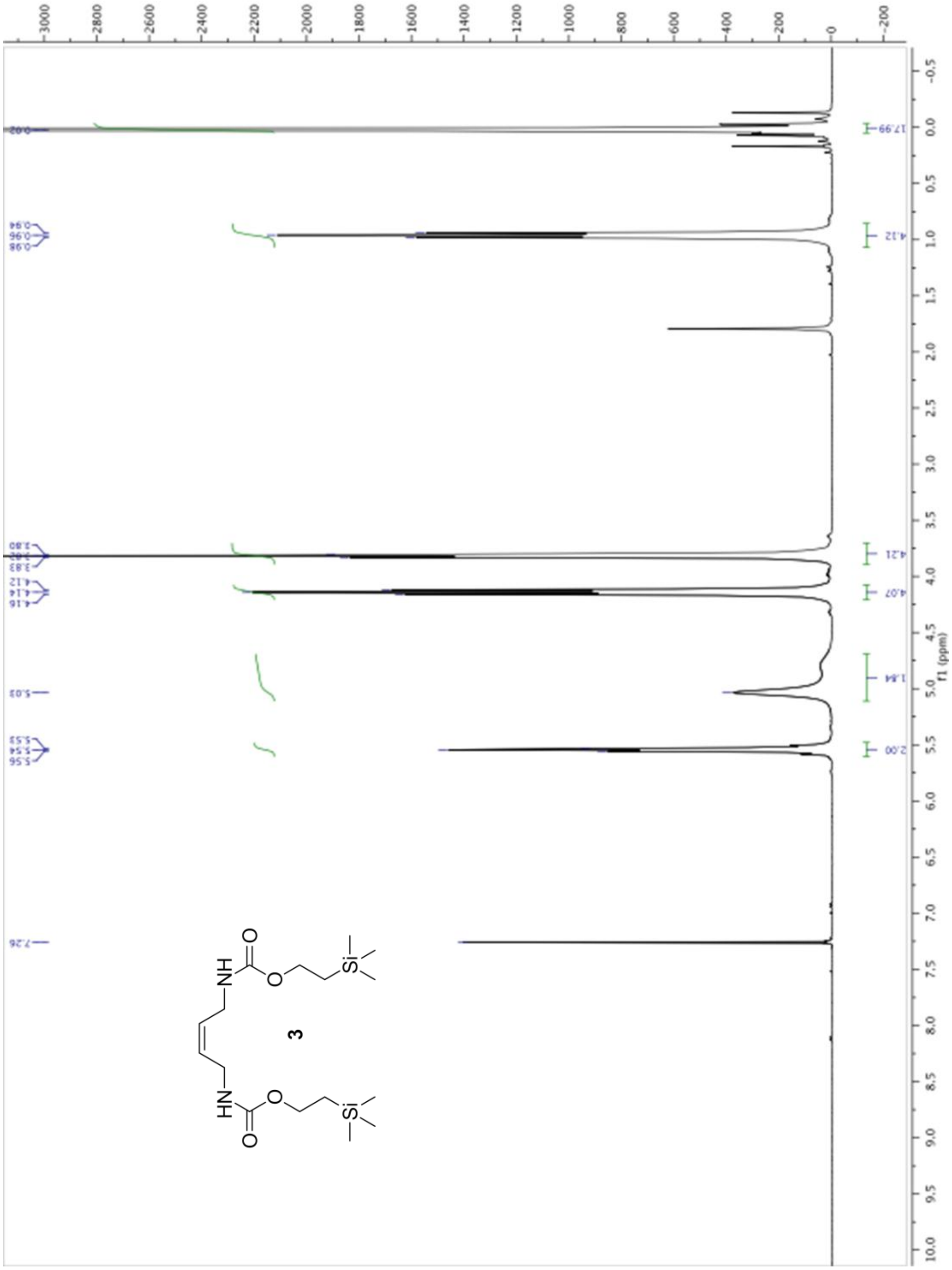

3
f1 (ppm)

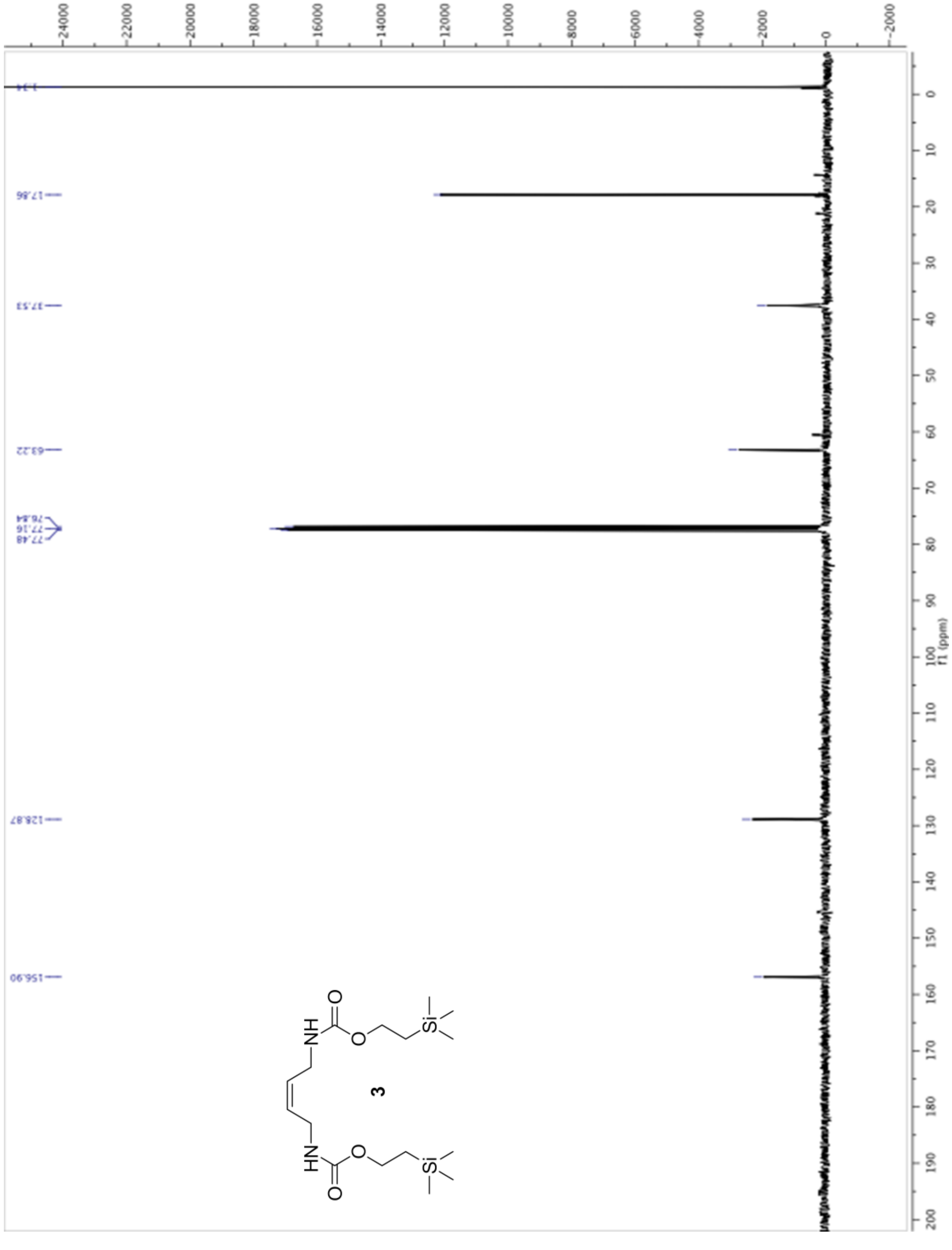
17.86
37.53
63.22
76.84
77.16
77.48
128.87
156.90
f1 (ppm)
3

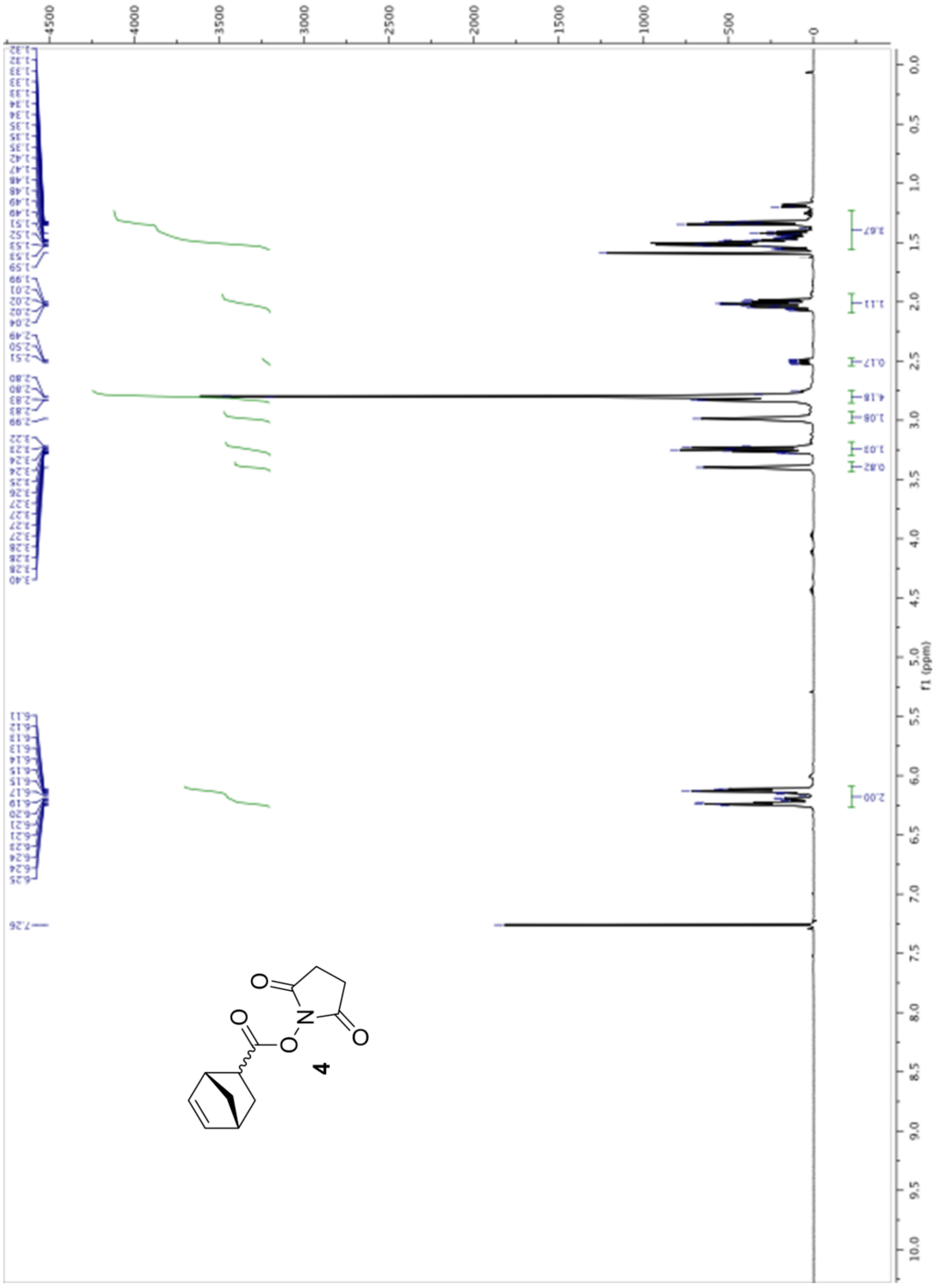

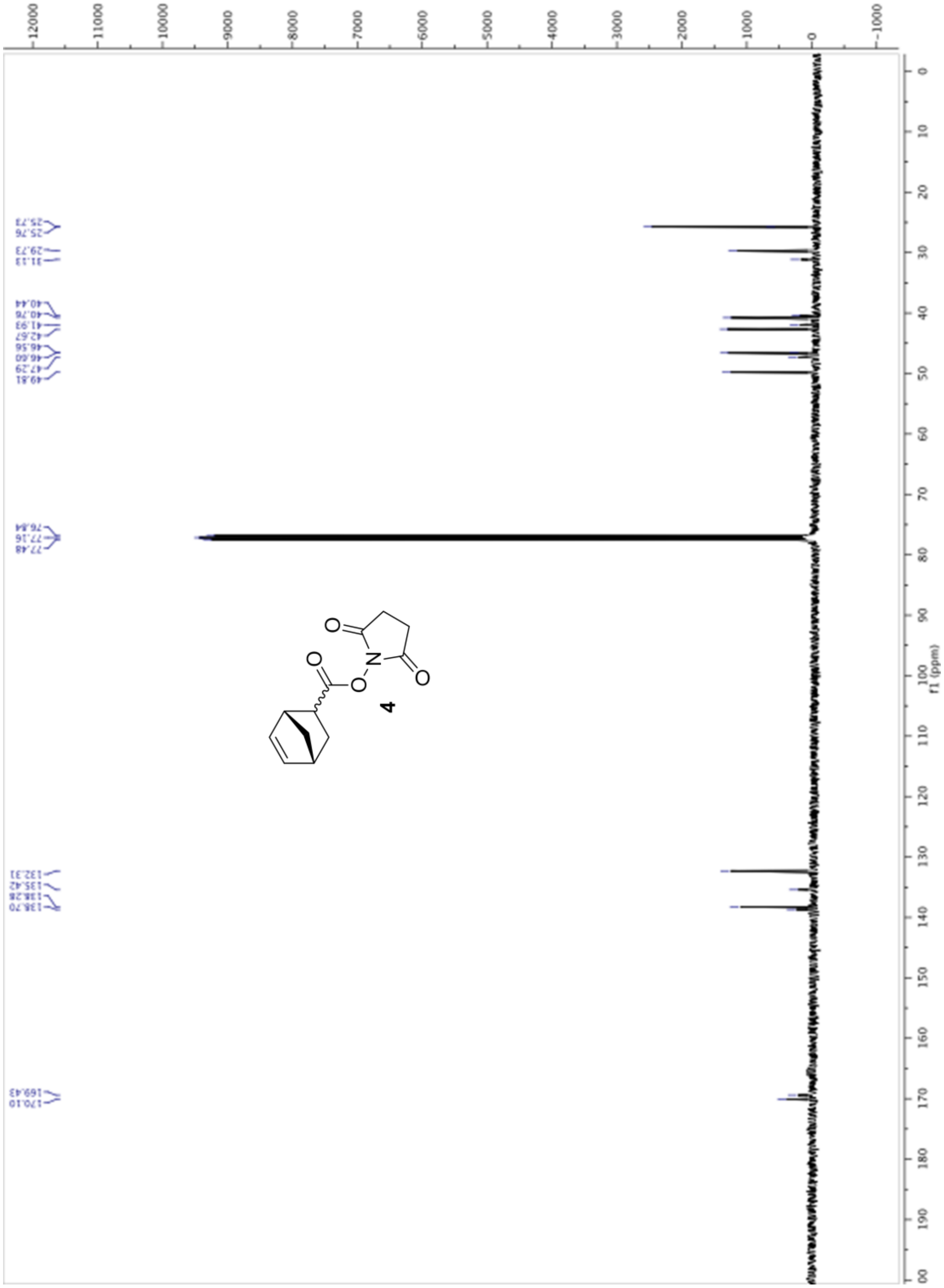
25.73
25.76
29.73
31.13
40.44
40.76
41.93
42.67
46.56
46.60
47.29
49.81
76.84
77.16
77.48
132.31
135.42
138.28
138.70
169.43
170.10
4
f1 (ppm)

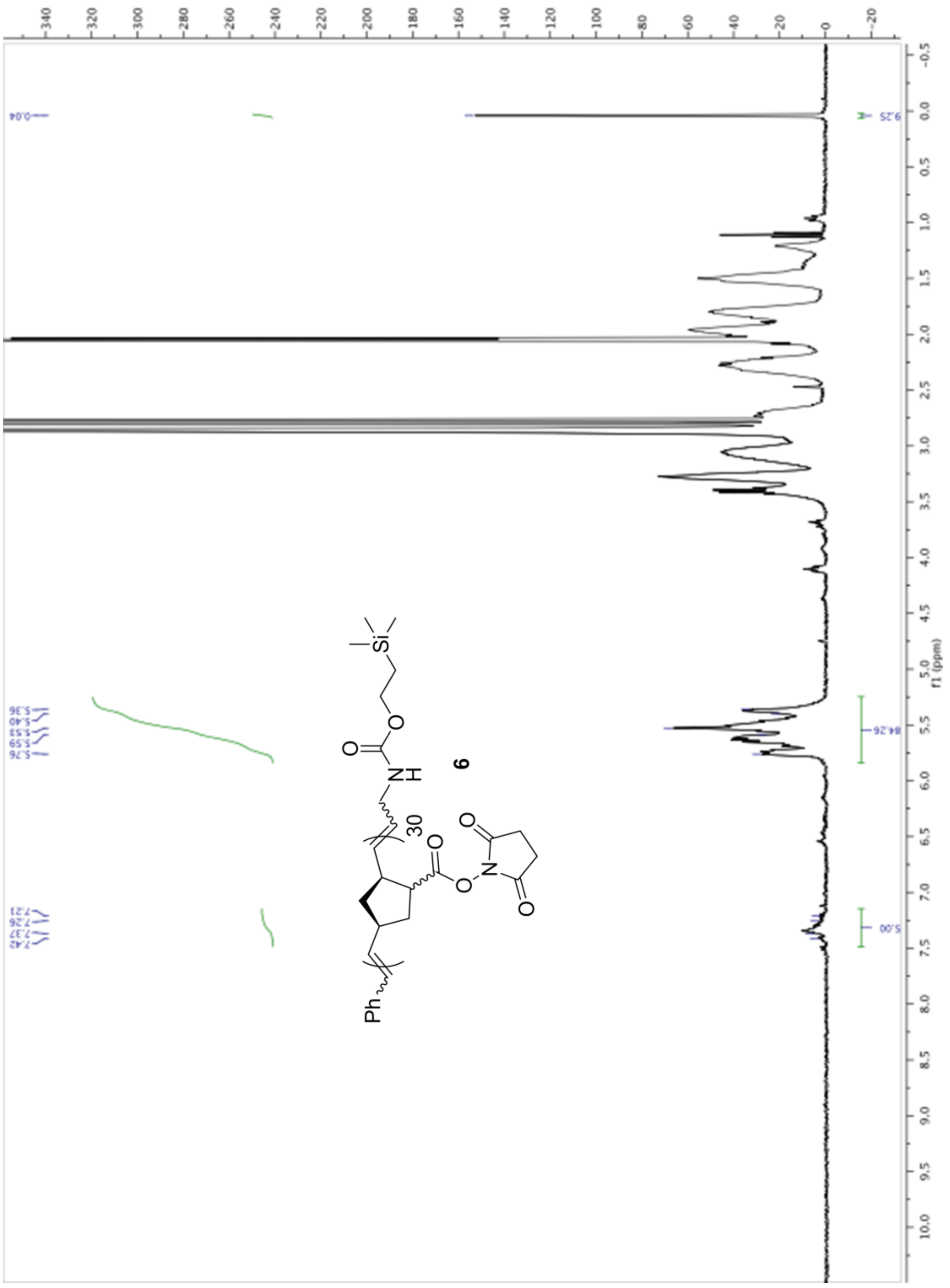
f1 (ppm)
Ph
30
Si
6

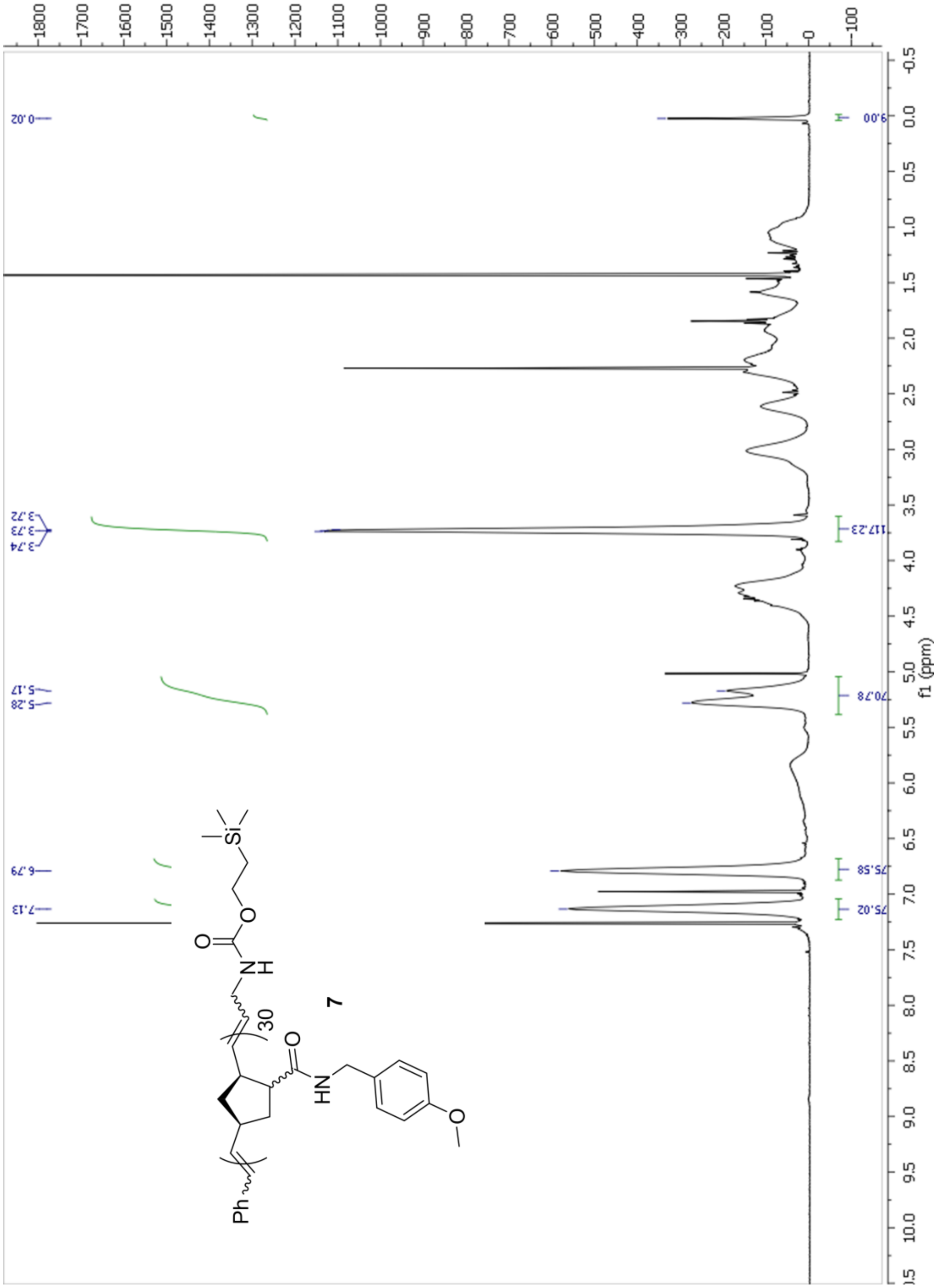

f1 (ppm)
7
Ph
Si
30
HN
O

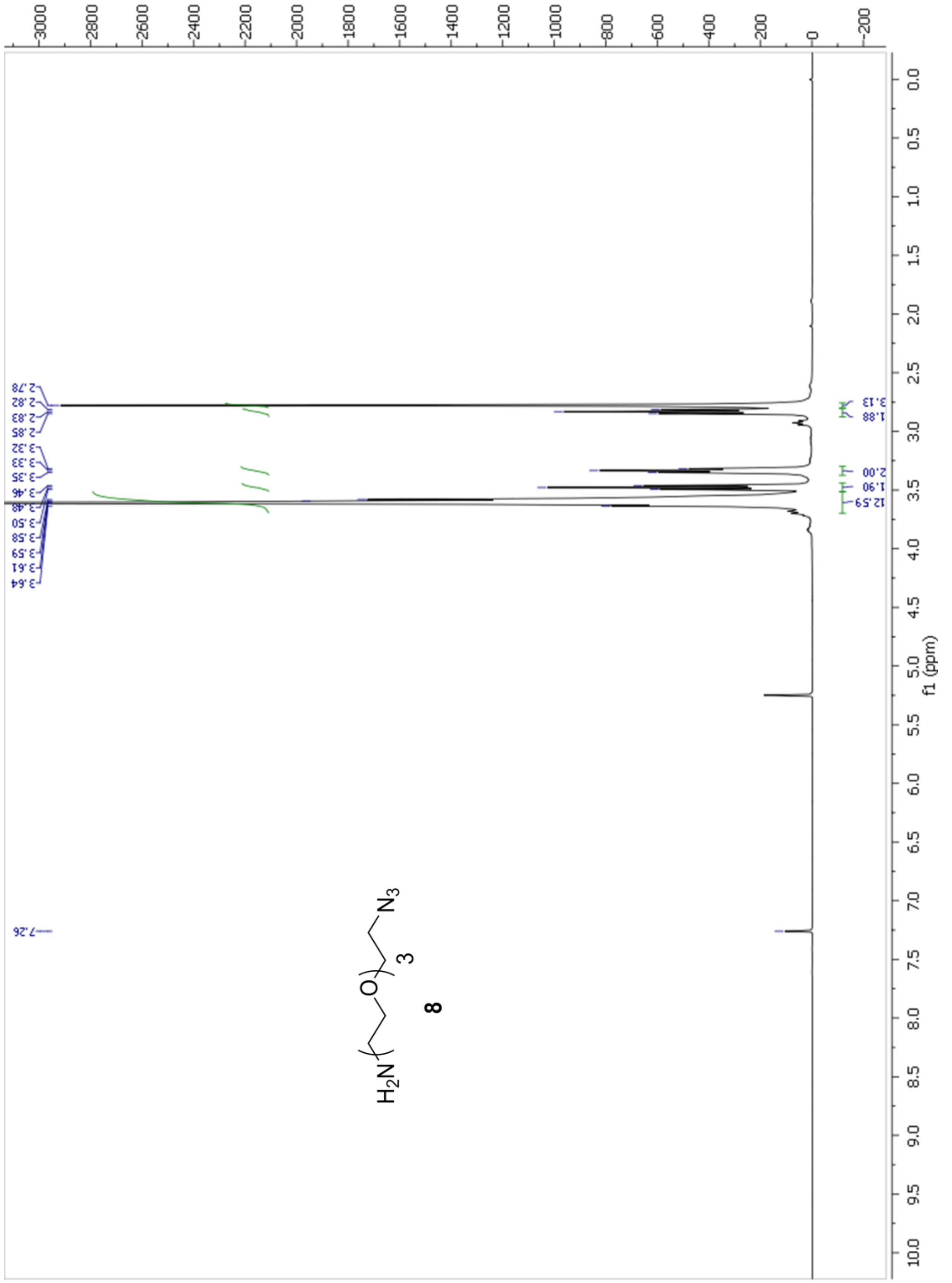
f1 (ppm)
8

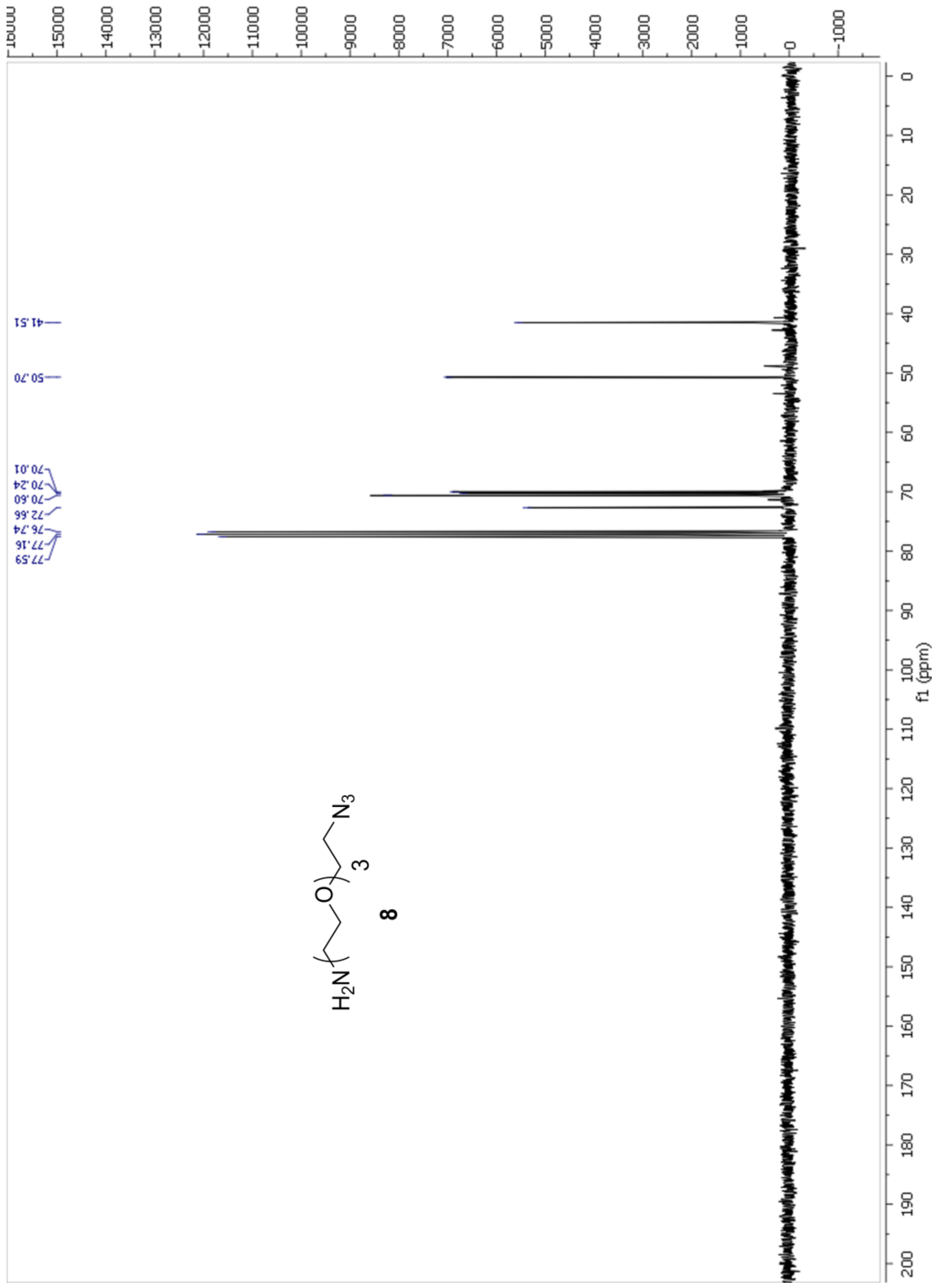
41.51
50.70
70.01
70.24
70.60
72.66
76.74
77.16
77.59
f1 (ppm)
8

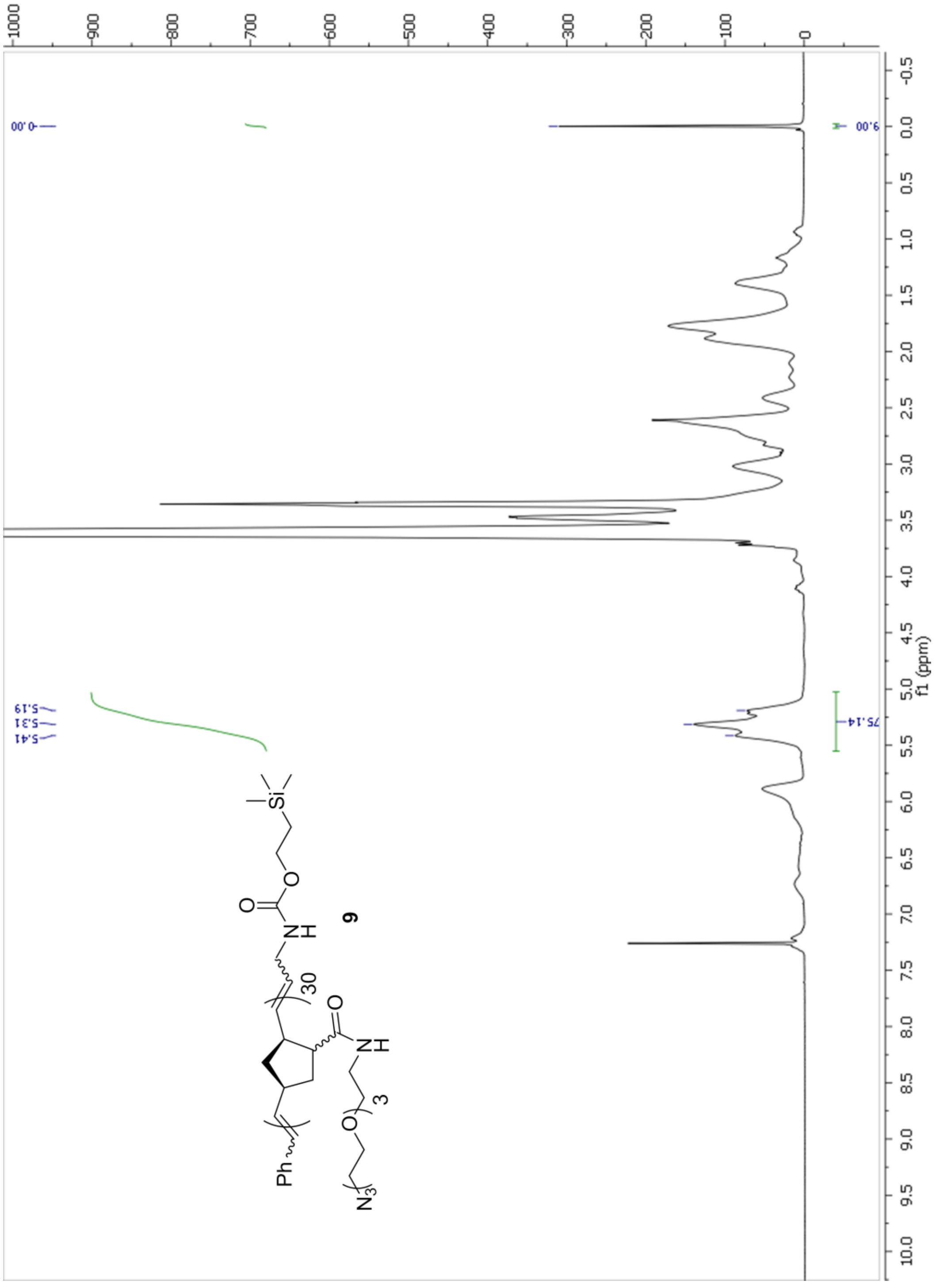

f1 (ppm)
0.00
5.19
5.31
5.41
9.00
75.14
9

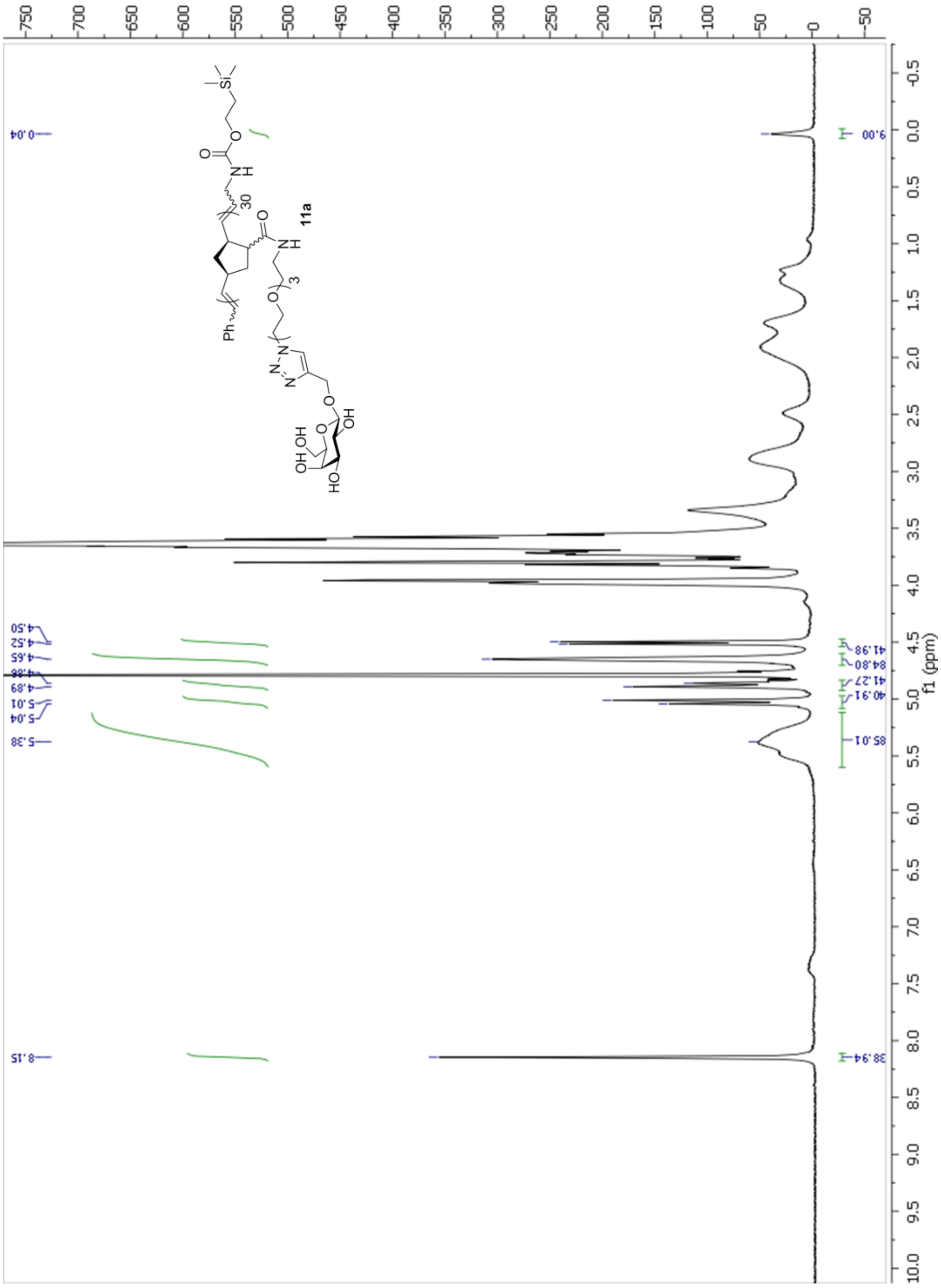

11a
Ph
30
3
f1 (ppm)
0.04
4.50
4.52
4.65
4.86
4.89
5.01
5.04
5.38
8.15
9.00
41.98
84.80
41.27
40.91
85.01
38.94

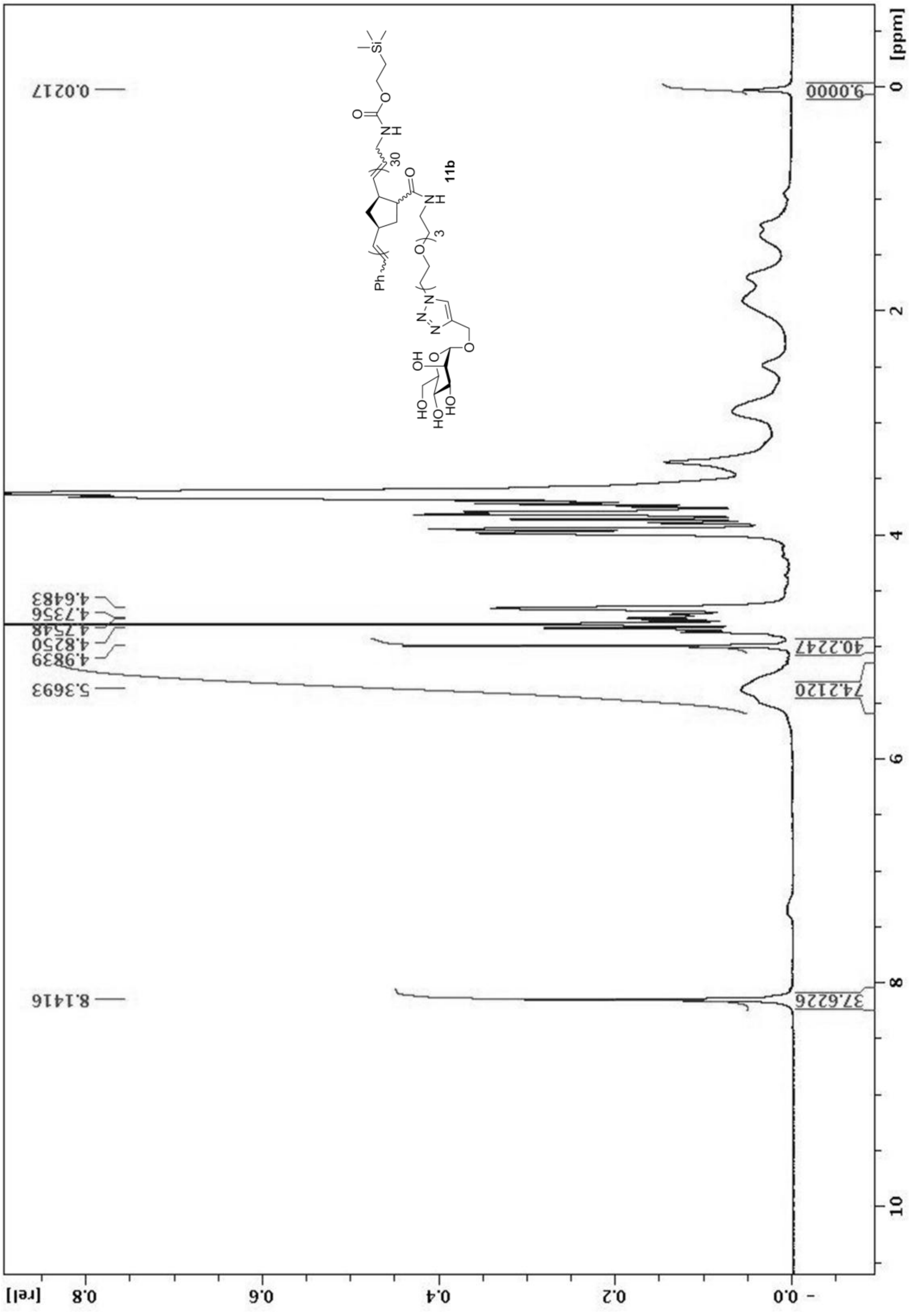

11b
0.0217
4.6483
4.7356
4.7548
4.8250
4.9839
5.3693
8.1416
9.0000
40.2247
74.2120
37.6226
[ppm]
[rel]

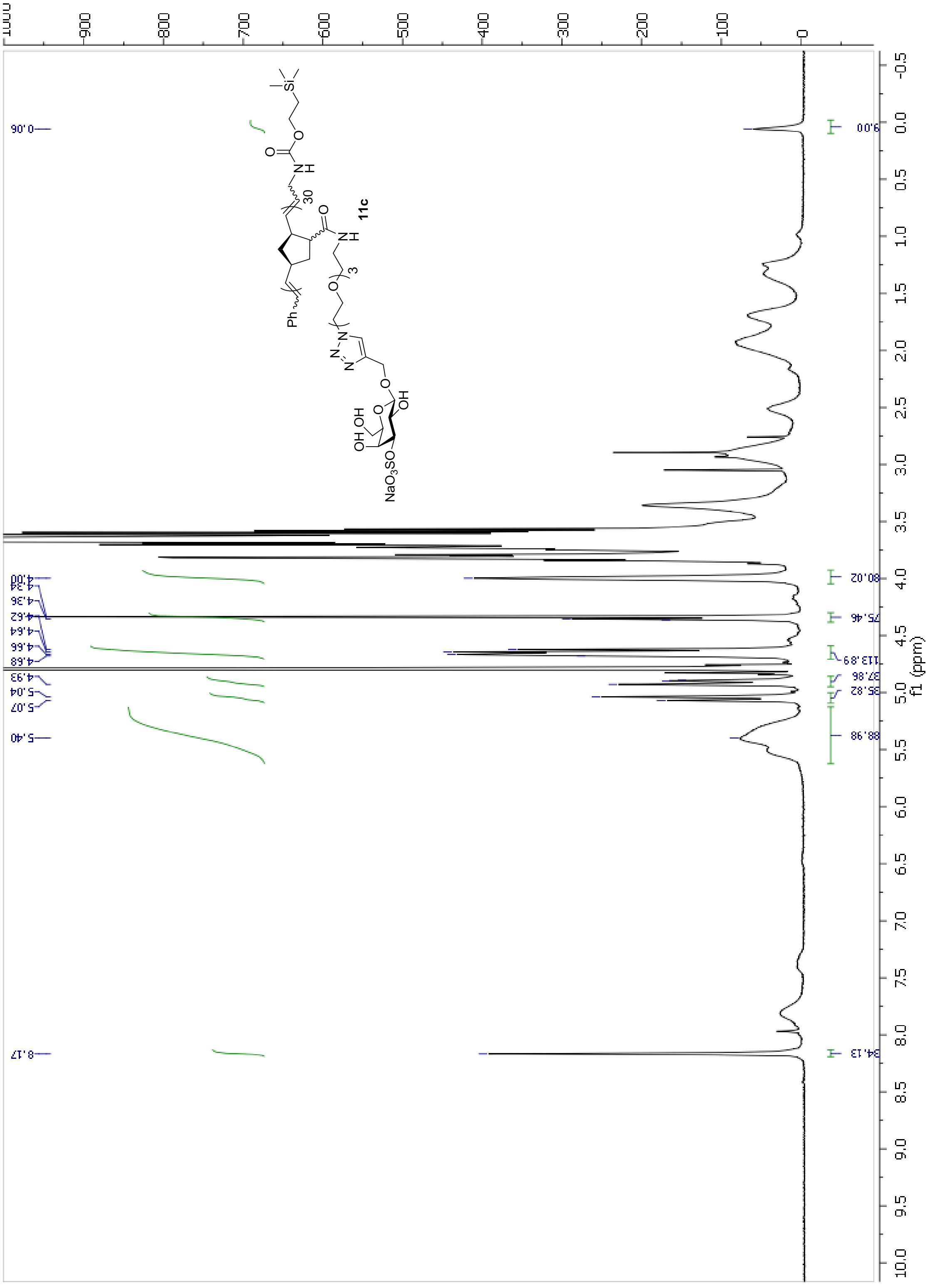

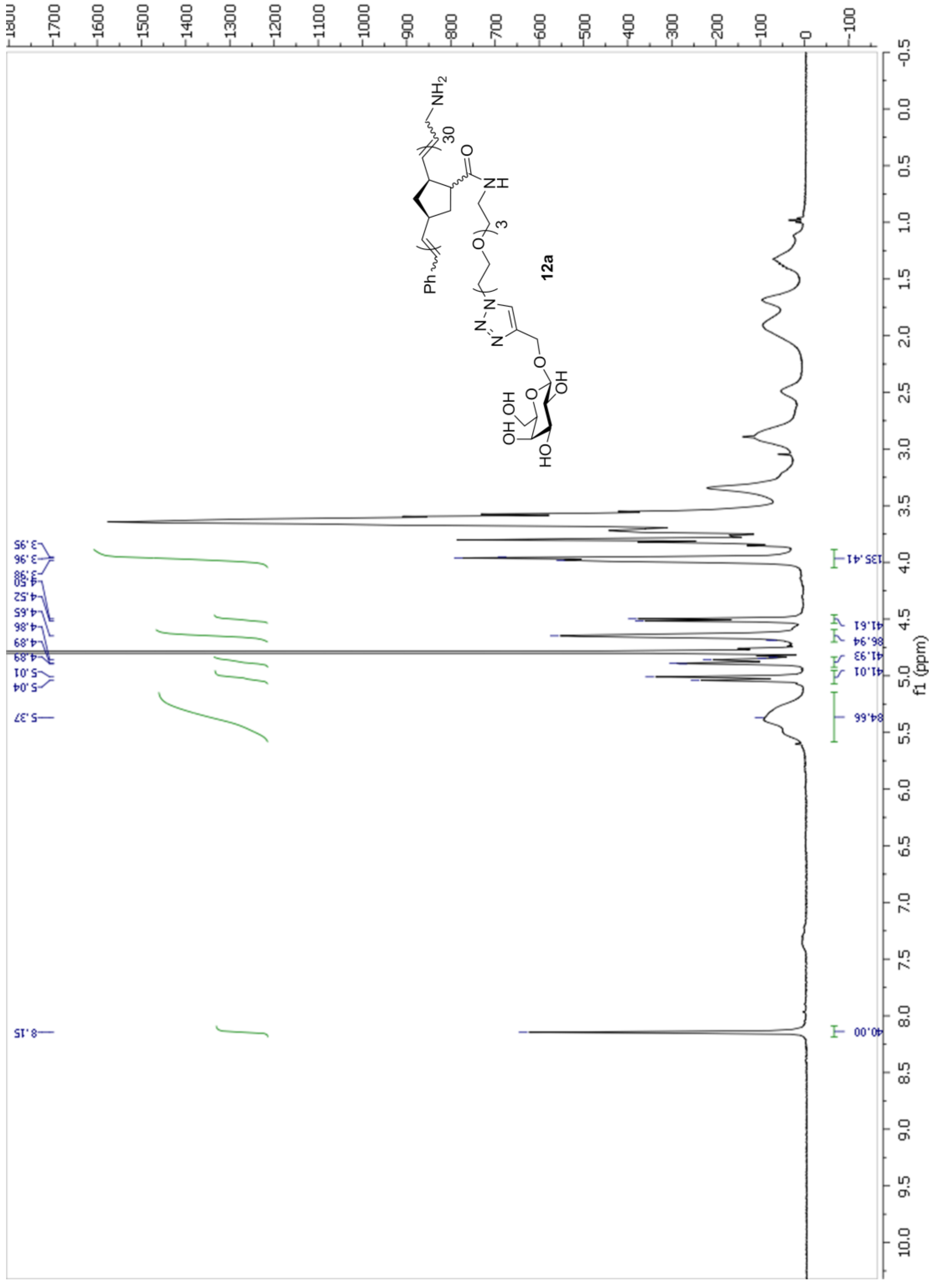
12a
f1 (ppm)

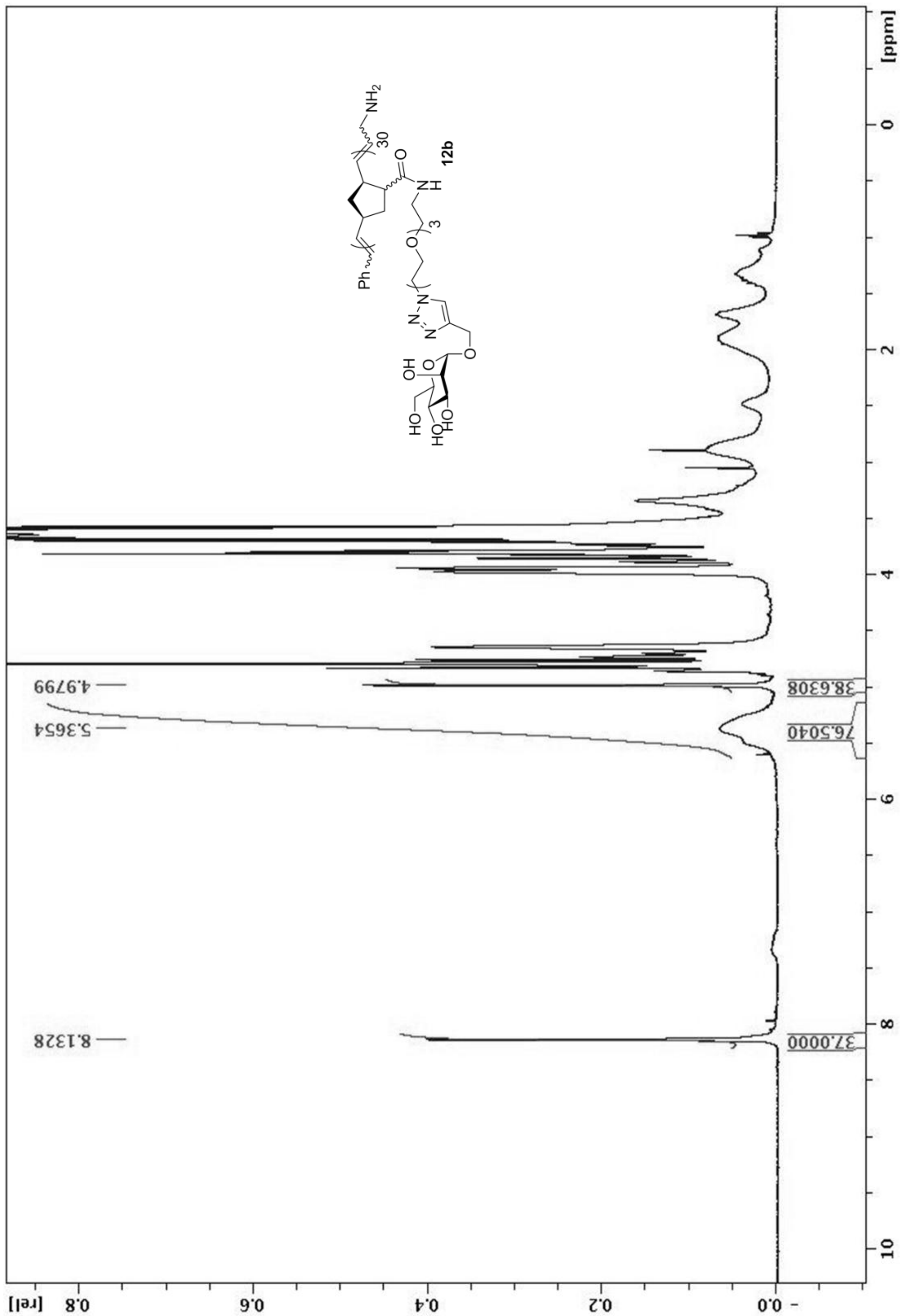
12b
8.1328
5.3654
4.9799
37.0000
76.5040
38.6308
[ppm]
[rel]

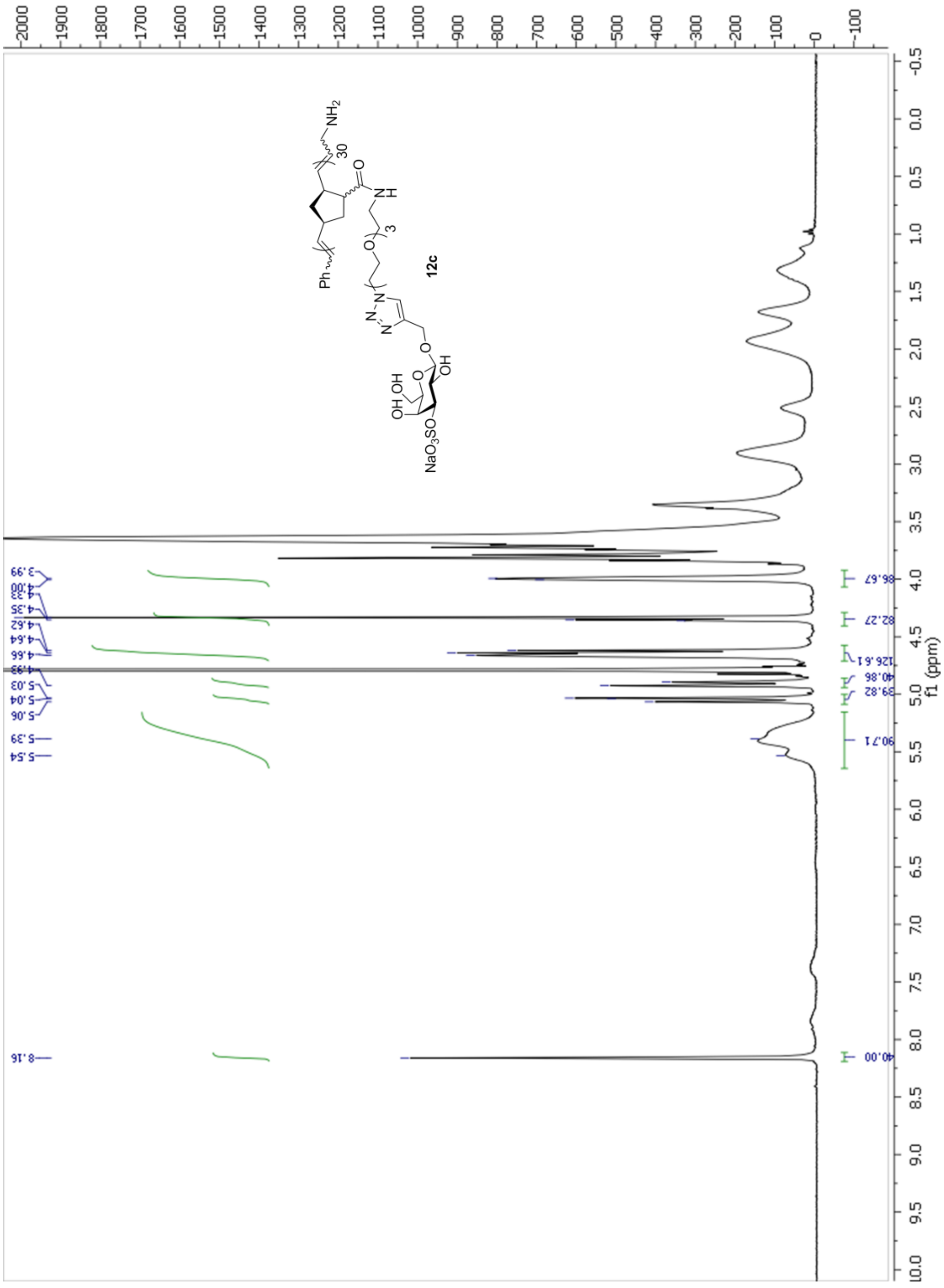
12c
f1 (ppm)

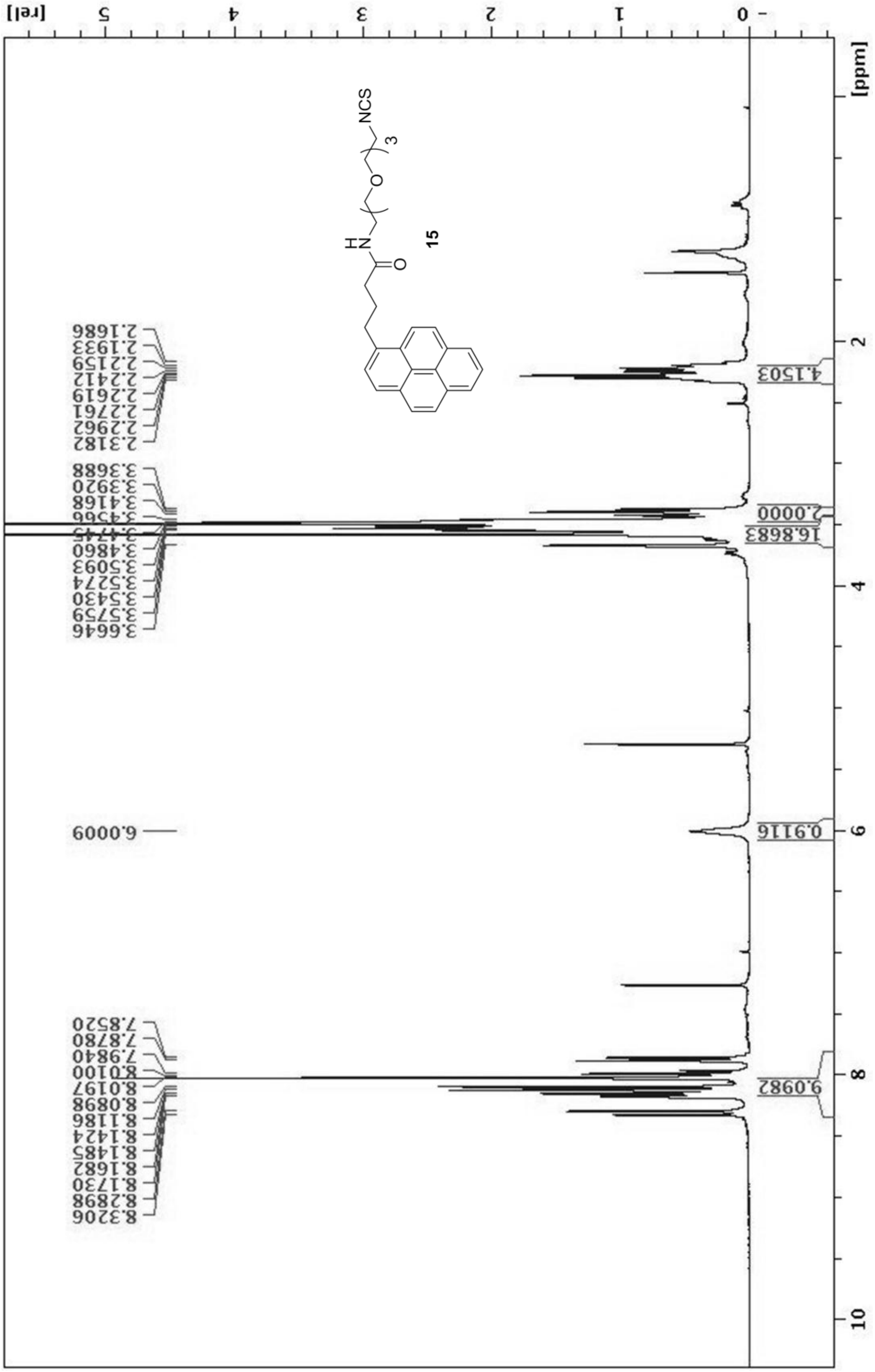
2.1686
2.1933
2.2159
2.2412
2.2619
2.2761
2.2962
2.3182
3.3688
3.3920
3.4168
3.4366
3.4745
3.4860
3.5093
3.5274
3.5430
3.5759
3.6646
6.0009
7.8520
7.8780
7.9840
8.0100
8.0197
8.0898
8.1186
8.1424
8.1485
8.1682
8.1730
8.2898
8.3206
4.1503
2.0000
16.8683
0.9116
9.0982
NCS
15
[ppm]
[rel]

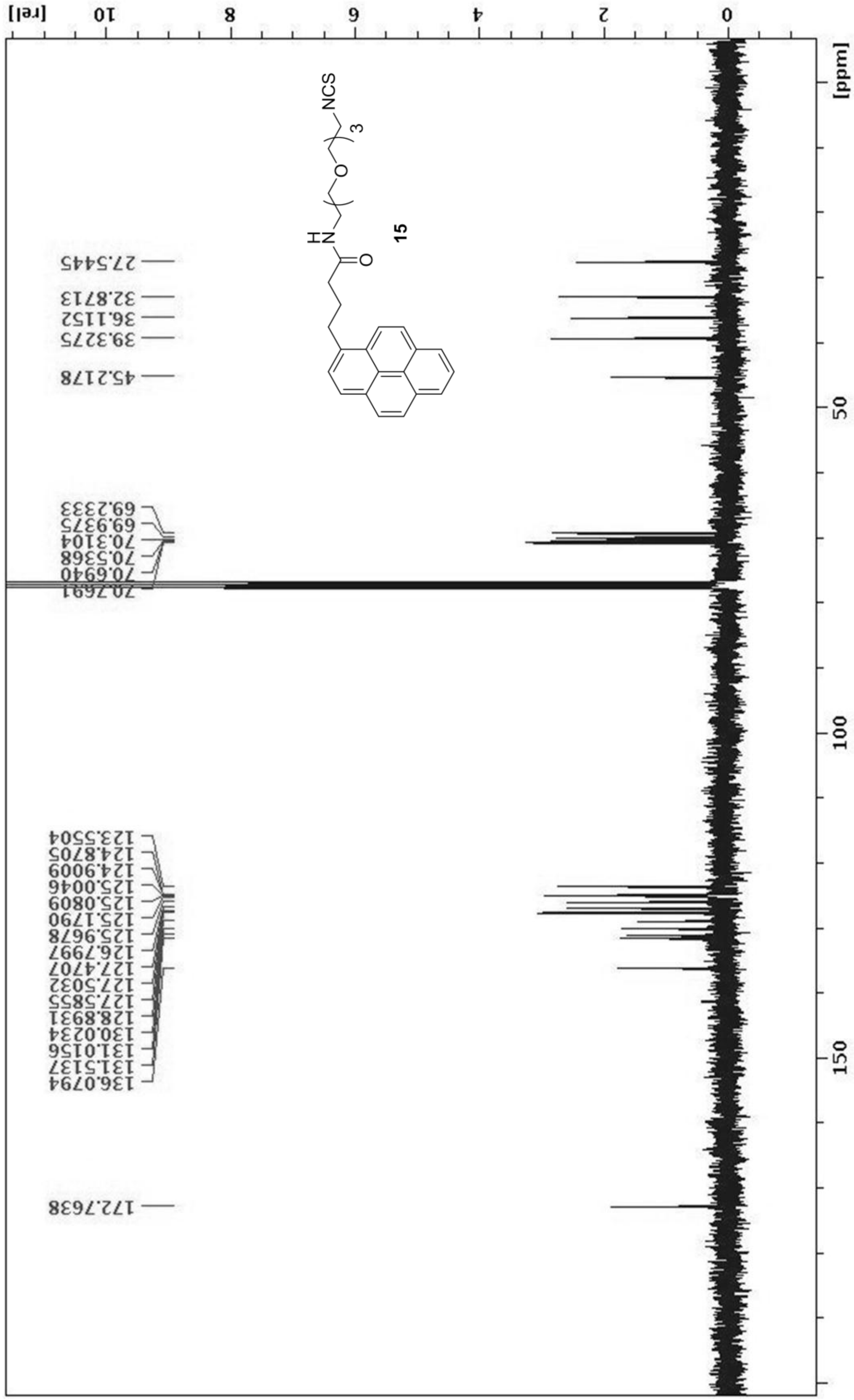

27.5445
32.8713
36.1152
39.3275
45.2178
69.2333
69.9375
70.3104
70.5368
70.6940
70.7691
123.5504
124.8705
124.9009
125.0046
125.0809
125.1790
125.9678
126.7997
127.4707
127.5032
127.5855
128.8931
130.0234
131.0156
131.5137
136.0794
172.7638
15
NCS
[ppm]
[rel]
